# Development of photoreactive demineralized bone matrix 3D printing colloidal inks for bone tissue engineering

**DOI:** 10.1093/rb/rbad090

**Published:** 2023-10-19

**Authors:** Katie J Hogan, Hayriye Öztatlı, Marissa R Perez, Sophia Si, Reyhan Umurhan, Elysa Jui, Ziwen Wang, Emily Y Jiang, Sa R Han, Mani Diba, K Jane Grande-Allen, Bora Garipcan, Antonios G Mikos

**Affiliations:** Department of Bioengineering, Rice University, MS-142, 6500 Main Street, Houston, TX 77030, USA; Baylor College of Medicine Medical Scientist Training Program, Houston, TX 77030, USA; Department of Bioengineering, Rice University, MS-142, 6500 Main Street, Houston, TX 77030, USA; Institute of Biomedical Engineering, Boğaziçi University, İstanbul, 34684, Turkey; Department of Bioengineering, Rice University, MS-142, 6500 Main Street, Houston, TX 77030, USA; Department of Bioengineering, Rice University, MS-142, 6500 Main Street, Houston, TX 77030, USA; Department of Bioengineering, Rice University, MS-142, 6500 Main Street, Houston, TX 77030, USA; Department of Bioengineering, Rice University, MS-142, 6500 Main Street, Houston, TX 77030, USA; Department of Bioengineering, Rice University, MS-142, 6500 Main Street, Houston, TX 77030, USA; Department of Bioengineering, Rice University, MS-142, 6500 Main Street, Houston, TX 77030, USA; Department of Bioengineering, Rice University, MS-142, 6500 Main Street, Houston, TX 77030, USA; Department of Bioengineering, Rice University, MS-142, 6500 Main Street, Houston, TX 77030, USA; Department of Bioengineering, Rice University, MS-142, 6500 Main Street, Houston, TX 77030, USA; Institute of Biomedical Engineering, Boğaziçi University, İstanbul, 34684, Turkey; Department of Bioengineering, Rice University, MS-142, 6500 Main Street, Houston, TX 77030, USA

**Keywords:** demineralized bone matrix, colloidal hydrogels, 3D printing, bone tissue engineering

## Abstract

Demineralized bone matrix (DBM) has been widely used clinically for dental, craniofacial and skeletal bone repair, as an osteoinductive and osteoconductive material. 3D printing (3DP) enables the creation of bone tissue engineering scaffolds with complex geometries and porosity. Photoreactive methacryloylated gelatin nanoparticles (GNP-MAs) 3DP inks have been developed, which display gel-like behavior for high print fidelity and are capable of post-printing photocrosslinking for control of scaffold swelling and degradation. Here, novel DBM nanoparticles (DBM-NPs, ∼400 nm) were fabricated and characterized prior to incorporation in 3DP inks. The objectives of this study were to determine how these DBM-NPs would influence the printability of composite colloidal 3DP inks, assess the impact of ultraviolet (UV) crosslinking on 3DP scaffold swelling and degradation and evaluate the osteogenic potential of DBM-NP-containing composite colloidal scaffolds. The addition of methacryloylated DBM-NPs (DBM-NP-MAs) to composite colloidal inks (100:0, 95:5 and 75:25 GNP-MA:DBM-NP-MA) did not significantly impact the rheological properties associated with printability, such as viscosity and shear recovery or photocrosslinking. UV crosslinking with a UV dosage of 3 J/cm^2^ directly impacted the rate of 3DP scaffold swelling for all GNP-MA:DBM-NP-MA ratios with an ∼40% greater increase in scaffold area and pore area in uncrosslinked versus photocrosslinked scaffolds over 21 days in phosphate-buffered saline (PBS). Likewise, degradation (hydrolytic and enzymatic) over 21 days for all DBM-NP-MA content groups was significantly decreased, ∼45% less in PBS and collagenase-containing PBS, in UV-crosslinked versus uncrosslinked groups. The incorporation of DBM-NP-MAs into scaffolds decreased mass loss compared to GNP-MA-only scaffolds during collagenase degradation. An *in vitro* osteogenic study with bone marrow-derived mesenchymal stem cells demonstrated osteoconductive properties of 3DP scaffolds for the DBM-NP-MA contents examined. The creation of photoreactive DBM-NP-MAs and their application in 3DP provide a platform for the development of ECM-derived colloidal materials and tailored control of biochemical cue presentation with broad tissue engineering applications.

## Graphical Abstract

**Figure:**
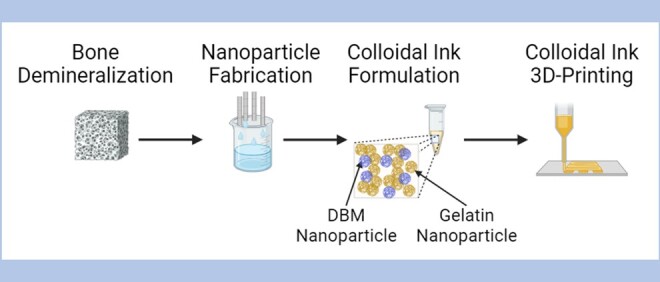

## Introduction

Craniofacial bone augmentation is often necessary as a result of tooth extraction, disease, trauma or tumor resection. Specifically, alveolar bone augmentation may be used to ensure sufficient bone depth and volume for dental implantation and assist in cleft palate repair [1]. The current clinical standard employs either autograft, which is associated with donor site morbidity, or barrier membranes for guided bone regeneration, which are associated with additional surgeries and high infection rates [2–4]. Likewise, orthopedic skeletal complications resulting from similar root causes require osteoconductive and osteoinductive void-filling materials for superior outcomes, as in the case of spinal fusions [5, 6]. Extracellular matrix (ECM)-derived materials, with their complexity and biocompatibility, have shown promise in the field of biomaterial-based tissue engineering due to their ability to mimic the tissue-specific microenvironment that supports tissue regeneration [7, 8]. One of the most common commercially available ECM-based biomaterials is demineralized bone matrix (DBM). Calcium content is suggested to be within the range of 1–6% for demineralized bone [9]. The remaining non-mineral component of bone has been found to contain predominantly collagen with additional non-collageneous proteins and osteoinductive proteins, including growth factors such as bone morphogenetic proteins [5, 6]. DBM has received clinical recognition for its osteoconductivity and osteoinductivity as a bone allograft to treat various dental, craniofacial and skeletal bone defects [10, 11]. With these advantages, DBM is used in roughly 50% of all allograft procedures in the USA [12]. However, lack of control over the material stiffness and swift degradation have caused difficulties in the application of DBM [13, 14]. DBM-only scaffolds without any form of crosslinking undergo rapid resorption after implantation, causing tissue collapse, which prevents bone formation [13]. Crosslinking systems have therefore been developed to improve the longevity of DBM implants and proved essential for tailoring DBM degradation and subsequent bioactive component release [15–18].

3D printing (3DP) is a fabrication technique that has gained interest for tissue engineering and regenerative medicine because it allows for precise control over scaffold size, shape and microarchitecture, key for the complex geometries encountered in craniofacial and orthopedic bone regeneration. 3DP has been used previously to print DBM in microparticle form within a poly(lactic-co-glycolic acid) ink [19–21]. However, printing without a synthetic carrier polymer would be preferable to increase the biocompatibility and bioactivity of the 3DP scaffolds. Recent work in our laboratory demonstrated the utility of a colloidal 3DP ink leveraging photoreactive gelatin nanoparticles (GNPs) for the fabrication of 3DP scaffolds with high print fidelity [22]. The use of photocrosslinking enabled the creation of a mechanically stable covalent interparticle network with designer control over scaffold swelling, degradation and biomolecule release [22]. However, gelatin lacks tissue-specific biochemical cues for tissue engineering applications, pointing to the need for additional bioactive components within a composite colloidal 3DP system.

In this study, DBM nanoparticles (DBM-NPs) were synthesized and methacryloylated [methacryloylated DBM-NPs (DBM-NP-MAs)] for use in colloidal composite 3DP inks with photoreactive methacryloylated GNPs (GNP-MAs). These inks combine the intrinsic bone regenerative cues found within DBM in photoreactive colloidal building blocks blended with GNP-MAs, formulated from ECM-derived gelatin, for increased printability and improved post-printing scaffold integrity. Here, we sought to determine the impact of DBM-NP inclusion on colloidal composite 3DP ink printability and the dose-dependent influence of DBM-NPs and ultraviolet (UV) crosslinking on 3DP scaffold swelling, degradation and osteogenic properties. To that end, DBM-NPs were synthesized and biochemically characterized. The rheological properties of GNP-MA and DBM-NP-MA composite colloidal inks were assessed as a function of incorporated DBM-NP-MA content. The physicochemical properties of GNP-MA:DBM-NP-MA scaffolds (henceforth referred to as GNP:DBM-NP scaffolds for the sake of simplicity since only methacryloylated nanoparticles were leveraged for 3DP) relative to DBM-NP-MA content and UV crosslinking were then analyzed via swelling and degradation studies, where changes in scaffold morphology and mass were evaluated over 21 days *in vitro*. Furthermore, the osteoinductive and osteoconductive effects of DBM-NP-MA content in UV-crosslinked 3DP scaffolds were evaluated in a 21-day *in vitro* study of mesenchymal stem cell osteogenesis. This study presents the creation of novel DBM-NP colloidal components for 3DP inks and confirms the osteoconductive properties of DBM-incorporating colloidal composite 3DP scaffolds.

## Materials and methods

### Demineralization of bone

The legs of 12- to 16-week-old female Sprague-Dawley SASCO rats were donated from Rice University in accordance with protocols approved by the Rice University Institutional Animal Care and Use Committee and the tibiae and femora harvested. Bone demineralization was adapted from previously established protocols [23]. Briefly, the soft tissue was removed from the bones, the ends were removed and bone marrow was flushed with phosphate-buffered saline (PBS) followed by an overnight wash in PBS + 1% antibiotic–antimycotic (anti–anti) at 4°C. Subsequently, the bones were lyophilized, ball milled and sieved to achieve particle sizes between 50 and 300 µm. These bone fragments were sequentially incubated in 95% ethanol for 16 h followed by ethyl ether for 6 h under agitation at 4°C. Following drying, the bone particles were demineralized by incubation in 0.6 M HCI at a weight ratio of 0.4 g/ml for 16 h at 4°C under gentle agitation. The resulting DBM was washed three times via centrifugation at 5000 rpm for 5 min and redispersion in Milli-Q water to remove residual organic solvents and the acidic supernatant. The DBM was frozen at −80°C, lyophilized and then stored at −20°C for further experiments.

### Synthesis and methacryloylation of nanoparticles

#### GNPs

GNPs were synthesized using a two-step desolvation method according to previously published methods [22]. An amount of 50 g of gelatin powder (Type B from bovine skin; bloom strength ∼225 g; Sigma-Aldrich, St. Louis, MO) was solubilized in 1 l of Milli-Q water at 50°C to which 1 l of acetone was added for the first desolvation step. The two phases were left at room temperature for 1 h without stirring, after which the supernatant was discarded and the precipitated gelatin (higher molecular weight fraction) was collected, resuspended in Milli-Q (0.5 l) and lyophilized prior to storage at −20°C. In the second desolvation step, 3.75 g of the precipitated gelatin was dissolved in 75 ml Milli-Q at 50°C and the pH was adjusted to 2.5. While this solution was stirred at 1200 rpm and 50°C, a multi-syringe infusion pump (Cole-Parmer, Vernon Hills, IL) was used to add 225 ml acetone dropwise via Teflon tubing (four tubes) at 12 ml/min. Once all the acetone was added, 555 µl of glutaraldehyde (25 wt%; Sigma-Aldrich, St. Louis, MO) was added to the nanoparticle suspension, which was then stirred overnight at room temperature. To consume any remaining aldehydes, guanidine hydrochloride (100 mM, 300 ml) was added the next day to the suspension with further stirring for 1 h. Then, the particles were filtered with a cell strainer (nylon, 40 µm mesh size; Fisher Scientific, Waltham, MA) followed by centrifugation (10 000 rpm for 1 h) and washing with Milli-Q three times. To methacryloylate the GNPs, GNPs were suspended at 10 mg/ml in 0.1 M CB buffer (91 mM sodium bicarbonate and 9 mM sodium carbonate), and the pH of the suspension was adjusted to 9 while stirring at 50°C. Methacrylic adhydride was then added dropwise at a ratio of 1.16 ml/g of GNPs and the reaction was allowed to continue for 1 h at 50°C and 800 rpm. The suspension was subsequently placed in SnakeSkin™ dialysis tubing (10 kDa molecular weight cutoff, Thermo Fisher Scientific, Rockford, IL) and dialyzed for 5 days against Milli-Q with daily Milli-Q exchanges. The GNP-MA suspension was then pH adjusted to 7.4, flash frozen and lyophilized prior to storage at −20°C until use.

#### DBM-NPs

DBM-NPs were synthesized using a two-step desolvation method modified from that for the GNPs described above [22]. To solubilize the DBM, 1 g of DBM was digested at 1 w/v% in 100 ml of 0.5 M acetic acid with 100 mg of pepsin (0.1 w/v%) for 96 h. Then, for the first desolvation, 100 ml of acetone was added, and the phases were left without agitation for 24 h at 4°C. After removing the cloudy supernatant, 50 ml of Milli-Q water was used to dilute the gelled precipitate and the pH was adjusted to 7 for lyophilization and storage at −20°C until the second desolvation. For nanoparticle formation, 100 mg of this DBM precipitate was dissolved in 50 ml of 0.5 M acetic acid and 70 ml of acetone was added dropwise at 1 ml/min using a multi-syringe pump and four pieces of Teflon tubing. To reduce turbulence, the stir rate for the suspension was gradually increased according to the volume of acetone added: 650 rpm for the first 10 min (to 40 ml acetone), then 700 rpm for 5 min (to 60 ml acetone) and 750 rpm for 2.5 min (to the full 70 ml acetone). Acetone addition increased opacity, indicating nanoparticle formation, and subsequently, 690 µl of glutaraldehyde (25 wt%) was added and the suspension was stirred at 4°C overnight. After this crosslinking, 36 ml of 0.1 M guanidine hydrochloride was added to quench the reaction and the suspension was stirred for 1 h at 4°C. A 40-µm cell strainer was used to filter the DBM-NPs, which were then centrifuged (10 000 rpm, 1.25 h) and washed with Milli-Q three times. DBM-NPs were suspended at a concentration of 1 mg/ml in 1 M CB buffer and a pH of 9 for methacryloylation. Methacrylic anhydride (1.16 ml/g DBM-NPs) was added dropwise to the suspension and allowed to react with the DBM-NPs for 2 h at room temperature. Dialysis was performed using dialysis tubing with a 10-kDa molecular weight cutoff against Milli-Q for 5 days with daily Milli-Q exchanges. The DBM-NP-MAs were then flash frozen and lyophilized prior to storage at −20°C until use.

### Biochemical characterization of DBM and DBM-NP

#### Scanning electron microscopy

Scanning electron microscopy (SEM) was performed to quantify nanoparticle diameter using a Quanta 400 system (Field Electron and Ion Company; Hillsboro, OR). GNPs, GNP-MAs, DBM-NPs and DBM-NP-MAs were dispersed in Milli-Q water. An SEM imaging stand was coated with 20 nm of gold and the nanoparticles were placed on top, flash frozen and then lyophilized. Thereafter, a 5-nm layer of gold was deposited on the lyophilized nanoparticles prior to imaging. This method produced a monolayer of particles to facilitate more representative estimation of size. ImageJ (version 1.53f) was used to quantify the dried diameters of GNPs, GNP-MAs, DBM-NPs and DBM-NP-MAs by manually measuring 100 nanoparticles per batch for three batches of each.

#### Biochemical assays

Native bone, DBM and DBM-NP-MA samples (10 mg/each) were solubilized in 1 ml of 0.5 M HCl solution containing 1 mg/ml pepsin for the analysis of DNA, total protein, collagen and sulfated glycosaminoglycan (sGAG) content and 1 ml of 0.25 M acetic acid solution containing 1 mg/ml of pepsin for calcium content by gently shaking at 37°C for 2 days [24, 25]. The biochemical component content of the native bone, DBM and DBM-NP-MA samples was determined by using a Picogreen Kit (Quant-iT dsDNA High Sensitivity Assay Kit, Molecular Probes, Life Technologies, Foster City, CA) for total DNA content and a previously published modified hydroxyproline assay for the collagen content [26]. The dimethylmethylene blue (DMMB) assay was used to determine sGAG content as previously published [27]. The calcium Arsenazo III assay (Pointe Scientific, Medtest Inc., CA) was used to quantify calcium according to literature protocols [8]. Three samples were used for each experimental group.

#### Nuclear magnetic resonance

The chemical structure of DBM following the first desolvation step, DBM-NP, DBM-NP-MA, gelatin after its first desolvation step, GNP and GNP-MA samples was characterized with ^1^H nuclear magnetic resonance (^1^H NMR) spectrometer (Bruker Biospin, Billerica, MA) to identify the methacryloyl group content of each material and confirm the methacryloylation of DBM-NPs and GNPs. The samples were prepared as reported previously [22]. To that end, 10 mg of the ball-milled samples were initially dissolved in PBS containing 2 mg/ml collagenase and incubated at 37°C for 3 days with regular vortexing. After complete dissolution, the samples were centrifuged at 5000 rpm for 5 min to remove any undissolved material. The supernatant of samples was transferred into separate tubes, frozen at −80°C and then lyophilized. Then, dried samples were dissolved in deuterium oxide consisting of 0.05 (w/v)% trimethylsilyl propanoic acid (TMSP) at a concentration of 7.5 mg/ml and subsequently analyzed with an ^1^H NMR spectrometer. The degree of methacryloylation was determined as the molar amount of methacryloyl groups per milligram of samples based on the amount of TMSP as an internal reference compound. The total amount of methacryloyl groups was calculated according to the equations reported previously in the literature [28].

### Preparation of GNP and DBM-NP colloidal composite inks

All colloidal composite inks were produced with a total nanoparticle content of 20 w/v%. GNP-MA and DBM-NP-MA composite inks were created by combining nanoparticle types in varying ratios, 100:0, 95:5 and 75:25, referring to the ratio of GNP-MA to DBM-NP-MA dry mass (GNP:DBM-NP) in that 20 w/v% solid component of the ink. 0.5 w/v% Irgacure 2959 in PBS was added to the GNP-MAs and DBM-NP-MAs, creating a paste. This mixture was vigorously mixed with a spatula, centrifuged and stored at 4°C. Inks were mixed and centrifuged twice daily over a 72-h swelling period prior to use for rheological characterization and 3DP. While inks were centrifuged in preparation to bring the material into a pellet, all inks were manually mixed with a spatula prior to transition to a 3DP cartridge, resulting in a consistent distribution of the solvent phase (PBS) throughout the ink for printing. Inks were swollen and handled without exposure to light prior to use to prevent extraneous UV crosslinking.

### Characterization of colloidal composite inks

#### Rheological analysis of ink printability

Initial rheological evaluation of composite colloidal GNP:DBM-NP ink printability was performed at 25°C with a Discover Hybrid Rheometer (DHR-2, TA Instruments, Newcastle, DE) using a 20-mm stainless steel flat head geometry with an operating gap of 300 µm. The viscosity of the bioinks was measured during flow ramps with an increasing shear rate from 10 to 1000/s to determine the shear-thinning behavior of bioinks. To ascertain self-healing behavior, ink recovery of storage and loss moduli following exposure to high shear was evaluated using a three-step oscillation protocol. First, storage (*G*′) and loss (*G*″) moduli values for each ink composition were determined in an initial time sweep performed for 1 min, at constant strain and frequency of 1% and 1 Hz, respectively. The shear stress of the printing process was then mimicked by applying a strain ramp from 0.1% to 1000%. Lastly, the recovery of the inks after this destructive shear was investigated with a 5-min time sweep at a constant 1% strain and 1-Hz frequency, simulating the return to low shear after ink deposition on the print platform. Yield stress and strain were likewise determined from the step II strain sweep using the crossover point of the storage (*G*′) and loss (*G″*) moduli. All measurements were taken using three samples.

#### Rheological analysis of ink viscoelastic properties and UV reactivity

Viscoelastic properties and UV reactivity of the composite GNP:DBM-NP inks were similarly performed at room temperature, with a 20-mm stainless steel flat head geometry, and using a 300-µm operating gap, on a Discover Hybrid Rheometer. In addition, an OmniCure S2000 Spot UV Curing System (Lumen Dynamics Group Inc., Mississauga, ON, Canada) was connected to the rheometer UV Light Guide Accessory (TA Instruments, Newcastle, DE), allowing UV crosslinking of inks with a 365-nm primary peak. Using this accessory, inks were crosslinked with a UV intensity of 10 mW/cm^2^ for 5 min, and the operating gap was adjusted to 260 µm afterward to accommodate ink syneresis and establish firm contact with the geometry for subsequent measurements. A frequency sweep of 0.1–100 Hz was performed before and after ink UV crosslinking. All measurements were taken using three samples.

### 3DP of composite colloidal inks

The colloidal composite GNP:DBM-NP inks were 3DP using an extrusion-based BioAssemblyBot^®^ 3D printer (Advanced Solutions Life Sciences). Two-layer, 10 mm × 10 mm lattice scaffolds were produced using a 20-G needle, a print speed of 0.8 mm/s and print pressures ranging from 20 to 30 psi (138–207 kPa). A fiber spacing (center-to-center) of 2 mm was used, and the perpendicular layers were designed to have 25% height overlap using a 0.45-mm interlayer distance to encourage vertical fiber–fiber adherence. Post-printing UV crosslinking was achieved using a UV light flash box (Otoflash Post Curing Light Pulsing Unit, EnvisionTEC Inc., Gladbeck, Germany) with 230- to 410-nm wavelengths delivered at ∼60 mW/cm^2^ for 50 s (500 flashes, 3 J/cm^2^ total).

### Characterization of 3DP GNP:DBM-NP scaffolds

#### Evaluation of in vitro swelling behavior

GNP:DBM-NP scaffold swelling behavior was determined over 21 days in PBS. Scaffolds were placed in PBS + 1% anti–anti under agitation at 37°C with media changes at 1 and 3 days with twice weekly thereafter. Using an optical microscope (trinocular stereomicroscope, SM-4TZ-144 A; AmScope, Irvine, CA), scaffolds were imaged immediately after printing, UV crosslinking and lyophilization for storage and after 1 h, 1, 3, 7, 14 and 21 days of swelling. ImageJ was used to convert images to binary, after which a custom Python code in JupyterNotebook (found at https://github.com/mrp13/Image-Analysis-of-3D-Printed-Scaffolds-) was used to quantify scaffold total non-porous area and pore area at each time point. These values were then normalized for each scaffold to initial post-printing values of these metrics for comparison between groups. Three to four scaffolds per DBM-NP content and UV crosslinking group were used, depending on scaffold stability and the potential for accurate image analysis.

#### Evaluation of in vitro degradation

GNP:DBM-NP scaffold degradation characteristics were evaluated in PBS + 1% anti–anti with and without 400 ng/ml collagenase. This collagenase level was chosen due to its physiological relevance to the native wound healing environment and for comparison to previous literature [22, 29]. Lyophilized scaffolds were placed in the respective medium and degraded over 21 days under agitation at 37°C with media changes after 1 days, 3 days and twice weekly subsequently. Scaffolds were degraded for 3, 14 or 21 days. At the appropriate time points, scaffolds were washed to remove salts and lyophilized, and the dry mass was documented and compared to their initial mass.

### Evaluation of 3DP GNP:DBM-NP scaffold in vitro osteogenic potential

#### Scaffold cell seeding

Bone marrow-derived human mesenchymal stem cells (hbMSCs), purchased from RoosterBio (Lot # 3210264, sex: female, age: 20 years, CD34 and CD45 antigen: <10% positive, CD90 and CD166 antigen: >90% positive, Frederick, MD), were expanded in a basal growth medium of Advanced MEM (Invitrogen, Carlsbad, CA) supplemented with 10% fetal bovine serum (FBS), 1% GlutaMAX and 1% anti–anti. Due to degradation results showing scaffold instability over 21 days without UV crosslinking, only UV-crosslinked scaffolds were evaluated for osteogenic potential. Low (95:5 GNP:DBM-NP) and high (75:25 GNP:DBM-NP) DBM-content scaffolds were evaluated for osteogenic potential. Lyophilized 3DP scaffolds were sterilized in a 12-h ethylene oxide cycle (Anprolene AN74i; Anderson Sterilizers, Haw River, NC) and vented for 48 h followed by overnight swelling in PBS + 1% anti–anti prior to cell seeding. Scaffolds were placed in a 12-well plate, and each was seeded with 1.25 × 10^5^ hbMSCs. Subsequently, scaffolds were incubated in either basal growth media or osteogenic media (basal growth media with additional 50 mg/l ascorbic acid, 10^−8^ M dexamethasone and 10 mM β‐glycerol 2‐phosphate). Media was changed after 24 h and then twice weekly thereafter. Scaffolds were collected at 3, 14 and 21 days for analysis. Four scaffolds were used per group per time point.

#### Biochemical assays for osteogenic markers

At each time point, scaffolds for biochemical analyses were washed with PBS for 15 min at 37°C and then stored at −20°C until characterization. Subsequently, thawed samples were homogenized in sterile Milli-Q using a Qiagen TissueLyser II (Hilden, Germany) at 30/s for 5 min. For Arsenazo III analysis of calcium content, an aliquot of the homogenized hydrogel suspension was diluted 1:1 with 1 M acetic acid and incubated for 16 h at room temperature to free Ca^2+^ ions. A colorimetric Calcium Arsenazo III Kit (Pointe Scientific, Canton, MI) was then used to quantify Ca^2+^ content as previously published, with 30 µl of sample incubated with 75 µl of Arsenazo III solution for 10 min at room temperature followed by reading the absorbance at 562 nm on a plate reader (Powerwave x340 Microplate Reader, BioTek Instruments, VT) [8]. The remaining homogenized hydrogel suspension was combined 1:1 with a digestion buffer {2 mg/ml proteinase K, 20 μg/ml pepstatin A and 370 μg/ml iodoacetamide in tris-EDTA solution [12.11 mg/ml tris(hydroxymethyl aminomethane), 0.744 mg/ml EDTA, pH 7.6]} for further analysis, as previously published [8, 30]. These digested samples were used to determine DNA content and alkaline phosphatase (ALP) activity. DNA content was quantified via PicoGreen assay (Quant-iT™ 1X dsDNA Assay Kit, high sensitivity, Invitrogen, Carlsbad, CA) according to manufacturer instructions. ALP activity was assessed with an Alkaline Phosphatase Assay Kit (Sigma-Aldrich, St. Louis, MO) according to manufacturer specifications.

#### RT-qPCR for osteogenic markers

The expression of two key osteogenic markers (RUNX2 and OCN) was assessed at each time point using quantitative reverse transcription polymerase chain reaction (RT-qPCR) similar to previous literature [8, 31]. Scaffolds were washed with PBS for 15 min at 37°C and then stored in RNAProtect reagent at −80°C until characterization. For analysis, scaffolds were homogenized as above with a Qiagen TissueLyser II at 30/s for 5 min and centrifuged, and the supernatant was removed. RNA isolation was performed using an RNEasy Mini Plus kit (Qiagen, Germantown, MD) according to manufacturer instructions using genomic DNA and RNA columns. RNA purity was then assessed via a NanoDrop 2000 spectrophotometer (Thermo Fisher, Waltham, MA) and the RNA was stored at −80°C until further analysis. The purified RNA was used to synthesize complementary DNA (cDNA) using a High-Capacity cDNA Reverse Transcription Kit (Applied Biosystems) according to manufacturer specifications and stored at −20°C. Subsequently, with primers specific to human RUNX2 (probe ID: Hs01047973_m1) and OCN (probe ID: Hs01587814_g1), RT-qPCR was performed with iTaq™ Universal Probes Supermix (Bio-Rad Laboratories) according to manufacturer instructions. Fold changes of these genes were first normalized to GADPH and then to the 95:5 basal growth media group, which was hypothesized to contain and be exposed to the lowest number of osteogenic cues. Three to four samples were used per group, dependent on measurable expression levels.

### Statistics

All data are presented as mean ± standard deviation, represented graphically by error bars, unless otherwise specified. Differences in biochemical analyses and rheological properties were assessed via a one-way analysis of variance (ANOVA) test. For swelling, degradation and *in vitro* experiments, at each time point, a two-way ANOVA with *post hoc* Tukey’s honestly significant difference (HSD) test was performed. Statistical significance was determined by *P *<* *0.05. Shared letters indicate no significant difference between groups. Statistics were performed using GraphPad Prism 6.01 and JMP Pro 16.

## Results

### Characterization of DBM-NP and GNP colloidal components

**Figure 1.**
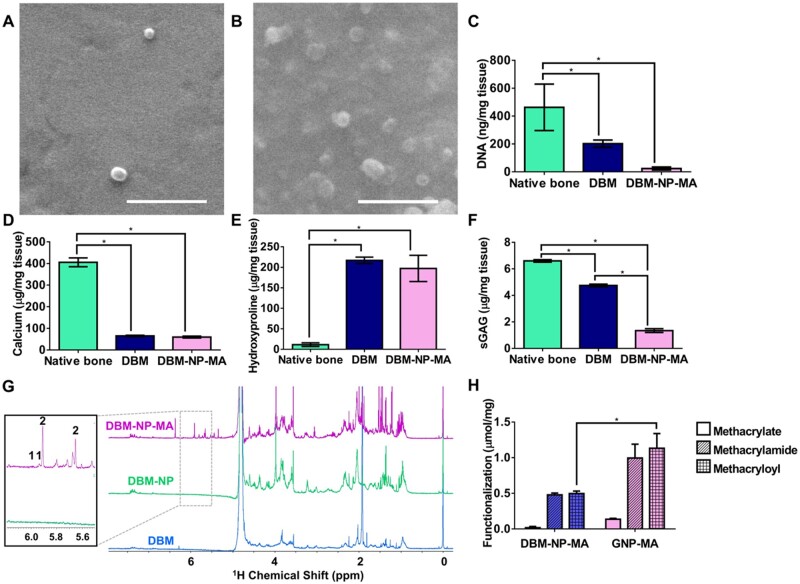
Characterization of DBM-NPs and DBM-NP-MAs. Representative SEM images of freeze-dried (**A**) DBM-NPs and (**B**) DBM-NP-MAs. Biochemical analysis and comparison of native bone, DBM and DBM-NPs. (**C**) DNA content quantified via PicoGreen assay. (**D**) Calcium content quantified via calcium assay. (**E**) Hydroxyproline content quantified via hydroxyproline assay. (**F**) sGAG content quantified via DMMB assay. (**G**) ^1^H NMR spectra for confirmation of methacryloylation: (1) methacrylate [5.9–6.2 ppm (CH_2_=C(CH_3_)COO–)] and (2) methacrylamide [5.6–5.9 ppm (CH_2_=C(CH_3_)CONH–] groups. (**H**) Quantified methacrylate, methacrylamide and total methacryloyl content for DBM-NP-MAs and GNP-MAs expressed as mmol per mg material. * indicates significant difference between groups (*n* = 3, *P* < 0.05). Scale bar = 500 nm.

First, as novel ECM-based nanoparticles, DBM-NPs were characterized. Using SEM (Fig. 1A and B), the freeze-dried diameter of DBM-NPs was determined to be 269 ± 44 nm and DBM-NP-MAs to be 405 ± 77 nm, which was significantly higher than that of DBM-NPs. Freeze-dried GNPs had a measured diameter of 98 ± 20 nm while GNP-MAs had a statistically similar diameter of 173 ± 54 nm. This difference in size between GNP-MAs and DBM-NP-MAs was attributed to the heterogeneous makeup of DBM. Then, biochemical components for native bone, DBM and DBM-NP-MAs were determined using a series of assays. DNA content (Fig. 1C) was quantified using a PicoGreen assay after DNA isolation. DBM samples had a DNA content of 202 ± 26 ng/mg, and further processing of DBM into DBM-NP-MAs resulted in a DNA content of 23.9 ± 10.9 ng/mg, both significantly lower than native bone with a DNA content of 463 ± 167 ng/mg. Calcium content was quantified with Calcium Arsenazo III assay (Fig. 1D). DBM and DBM-NP-MA samples had calcium contents of 65.1 ± 3.1 and 59.8 ± 4.0 μg/mg, respectively, significantly lower than the calcium content of 405 ± 21 μg/mg found in native bone. Collagen content was determined by the hydroxyproline assay (Fig. 1E). The collagen content in DBM was 217 ± 8 μg/mg, where it was markedly higher than the collagen found in native bone (11.4 ± 4.5 μg/mg). DBM-NP-MAs had a collagen content of 197 ± 32 μg/mg, which was not significantly different from DBM. As seen in Fig. 1F, sGAG content was quantified with DMMB assay. sGAG content in native bone was 6.6 ± 0.1 μg/mg and was significantly decreased in DBM (4.8 ± 0.1 μg/mg) and in DBM-NP-MAs (1.3 ± 0.1 μg/mg).

Figure 1G and H demonstrates the successful methacryloylation of DBM-NPs via NMR analysis. The spectrum shown in Fig. 1G shows newly formed peaks between 5.6 and 6.2 ppm specific to the formation of methacrylate [peaks labeled 1, 5.9–6.2 ppm (CH_2_=C(CH_3_)COO–), methacryloyl group reaction with hydroxyl groups] and methacrylamide [peaks labeled 2, 5.6–5.9 ppm (CH_2_=C(CH_3_)CONH–), methacryloyl group reaction with amine groups] modification for DBM-NP-MAs versus DBM-NPs. These peaks were also observed in GNP-MAs after methacryloylation compared to gelatin and GNPs (Supplementary Fig. S1). By integrating these observed peaks, the concentration of methacryloyl groups present in DBM-NPs was calculated to be 0.018 ± 0.017 and 0.479 ± 0.025 mmol of the methacrylate and methacrylamide groups, respectively, per milligram of material (Fig. 1H). GNP-MAs had concentrations of 0.137 ± 0.013 and 0.995 ± 0.194 mmol methacrylate and methacrylamide groups, respectively, per milligram of material.

### Rheological characterization of GNP:DBM-NP composite colloidal inks

**Figure 2.**
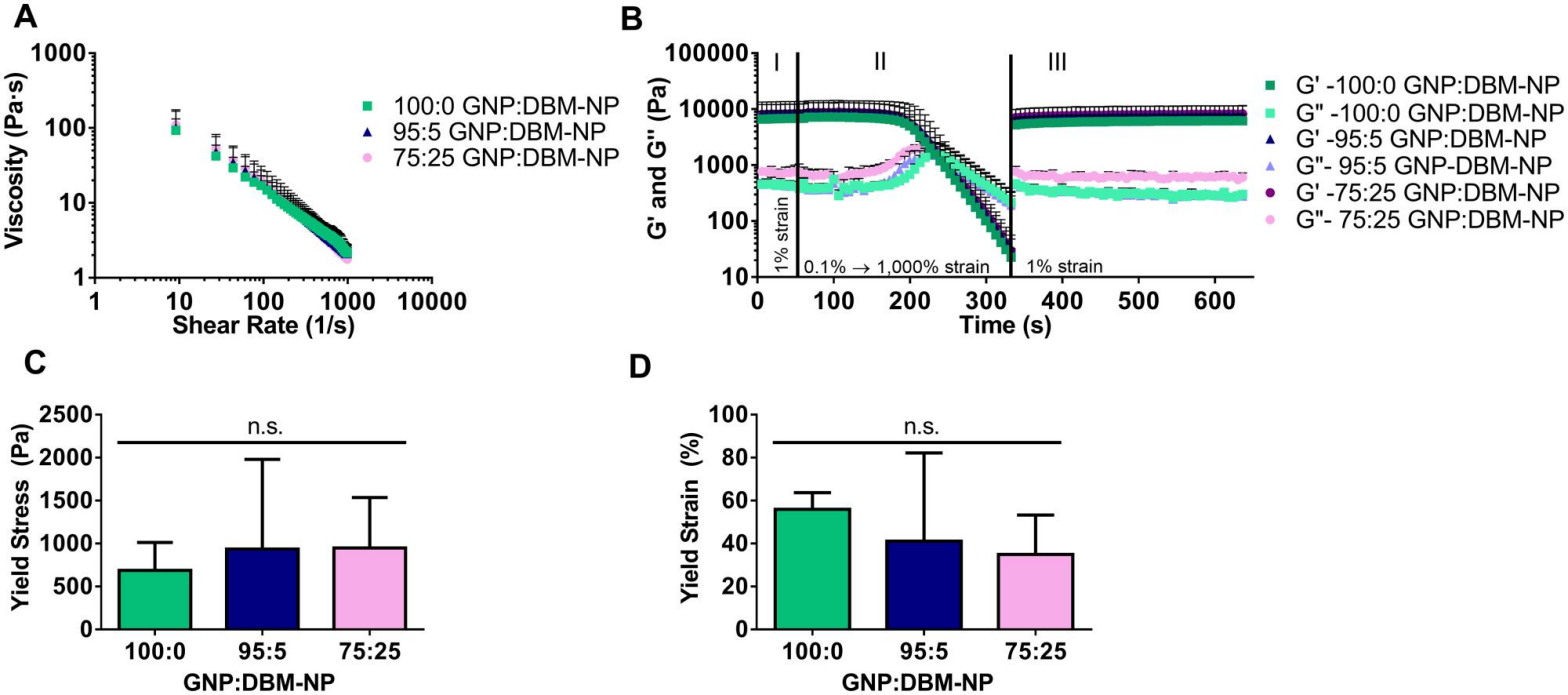
Rheological and printability assessment of colloidal inks with varying DBM-NP-MA contents. (**A**) Shear-thinning behavior evaluated with a flow ramp of shear rates from 0.1 to 1000/s. (**B**) Recovery or self-healing behavior measured with a three-step protocol: (I) time sweep with low strain (1%), (II) strain sweep from 0.1% to 1000% for network destruction and (III) time sweep low strain (1%) to observe return to baseline properties. (**C**) Yield stress and (**D**) strain quantified via the strain sweep (*n* = 3, *P* < 0.05). n.s. indicates non-significance.

To determine how the introduction of DBM-NP-MAs into GNP-MA colloidal inks would influence printability and colloidal stability, the rheological properties of GNP:DBM-NP ink compositions (100:0, 95:5 and 75:25 GNP:DBM-NP, with both nanoparticle types methacryloylated) were evaluated. All inks showed shear-thinning behavior with similar decreases in ink viscosity in response to increasing shear rate, confirming extrudability (Fig. 2A). Of note, the addition of DBM-NP-MAs did not impact the overall viscosity of the inks even in the 75:25 GNP:DBM-NP ink, the highest DBM-NP-MA content. Likewise, Fig. 2B shows the robust thixotropic recovery behavior of all ink compositions following the network disruption in the strain ramp. After this disruption, simulating the shear experienced during extrusion, and upon return to low strain, the storage and loss moduli of all inks immediately returned to the same order of magnitude of their initial values with storage moduli higher than loss moduli, behaving as solid gels once more. Yield stress (Fig. 2C) and strain (Fig. 2D), evaluated from the strain ramp data, also remained similar across the DBM-NP-MA content groups. This behavior demonstrates that DBM-NP-MAs may be incorporated within colloidal composite inks, here with GNP-MAs, without significantly impacting rheological properties and processes correlating to ink printability.

### UV crosslinking of GNP:DBM-NP composite colloidal inks

**Figure 3.**
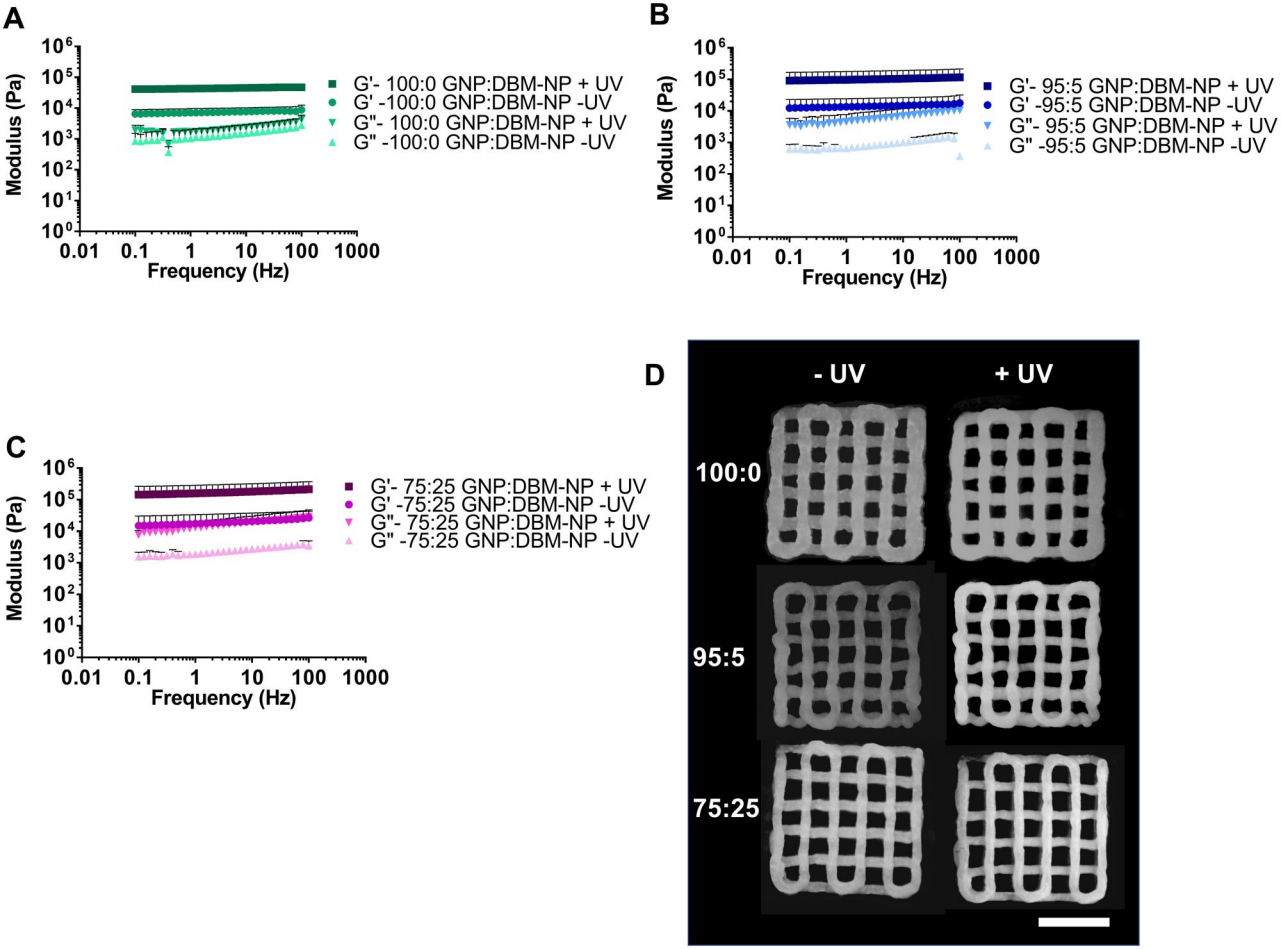
Composite GNP:DBM-NP ink UV crosslinking according to the DBM-NP-MA content in the presence and absence of UV exposure. Frequency sweeps from 0.1 to 100 Hz presenting storage (*G*′) and loss (*G*″) moduli for (**A**) 100:0 GNP:DBM-NP, (**B**) 95:5 GNP:DBM-NP and (**C**) 75:25 GNP:DBM-NP scaffolds before and after UV exposure. (**D**) Representative images of 3DP scaffolds before and after UV crosslinking with 3 J/cm^2^ (*n* = 3). Scale bar = 5 mm.

The UV crosslinking GNP:DBM-NP composite colloidal inks was evaluated using photorheometry. First, frequency sweeps of inks were recorded before and after exposure to UV light. As seen in Fig. 3A–C (100:0, 95:5 and 75:25 GNP:DBM-NP, respectively), all inks demonstrated frequency-independent gel-like behavior and an increase in both storage and loss moduli following UV exposure. Representative images of printed two-layer, 10 mm × 10 mm scaffolds are shown in Fig. 3D, showing the similarity in practical printability among the ink compositions. Additionally, image analysis showed no scaffold morphological differences (non-porous area and average pore area) before and after UV crosslinking (Supplementary Fig. S2).

### Swelling of 3DP GNP:DBM-NP scaffolds

**Figure 4.**
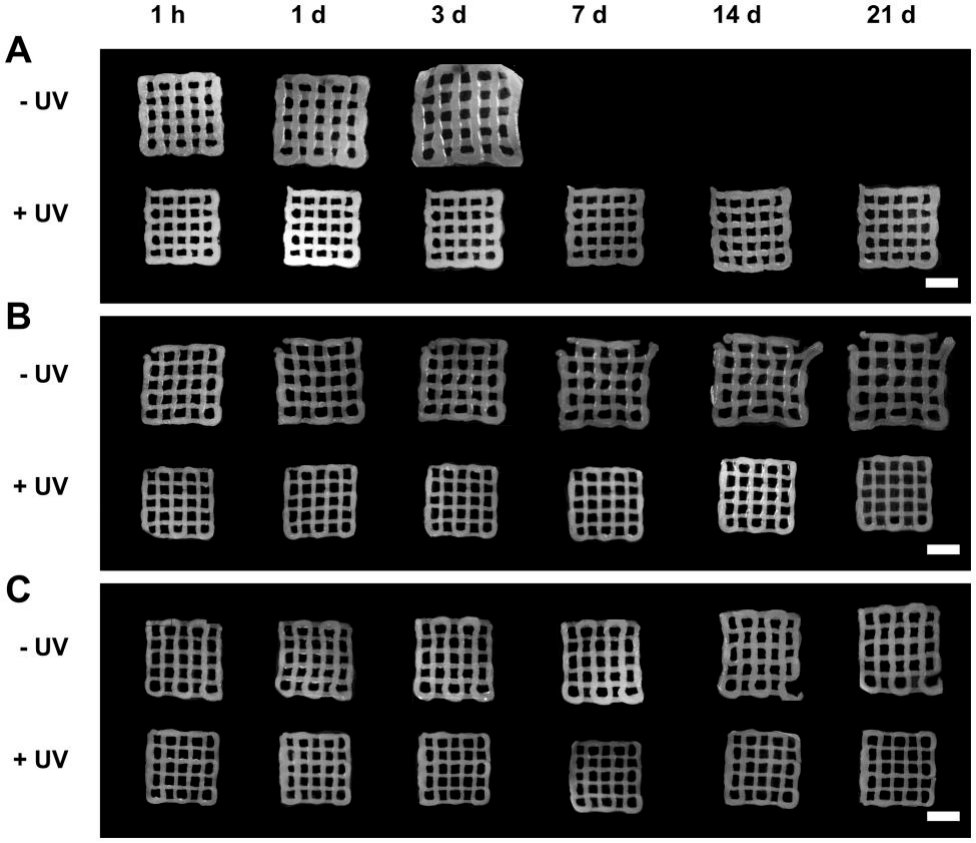
3DP GNP:DBM-NP scaffold swelling over time with varied DBM-NP-MA content and UV exposure for photocrosslinking. Representative images of (**A**) 100:0 GNP:DBM-NP, (**B**) 95:5 GNP:DBM-NP, and (**C**) 75:25 GNP:DBM-NP scaffolds. Empty spaces indicate scaffold dissolution precluding image acquisition. Scale bar = 5 mm.

**Figure 5.**
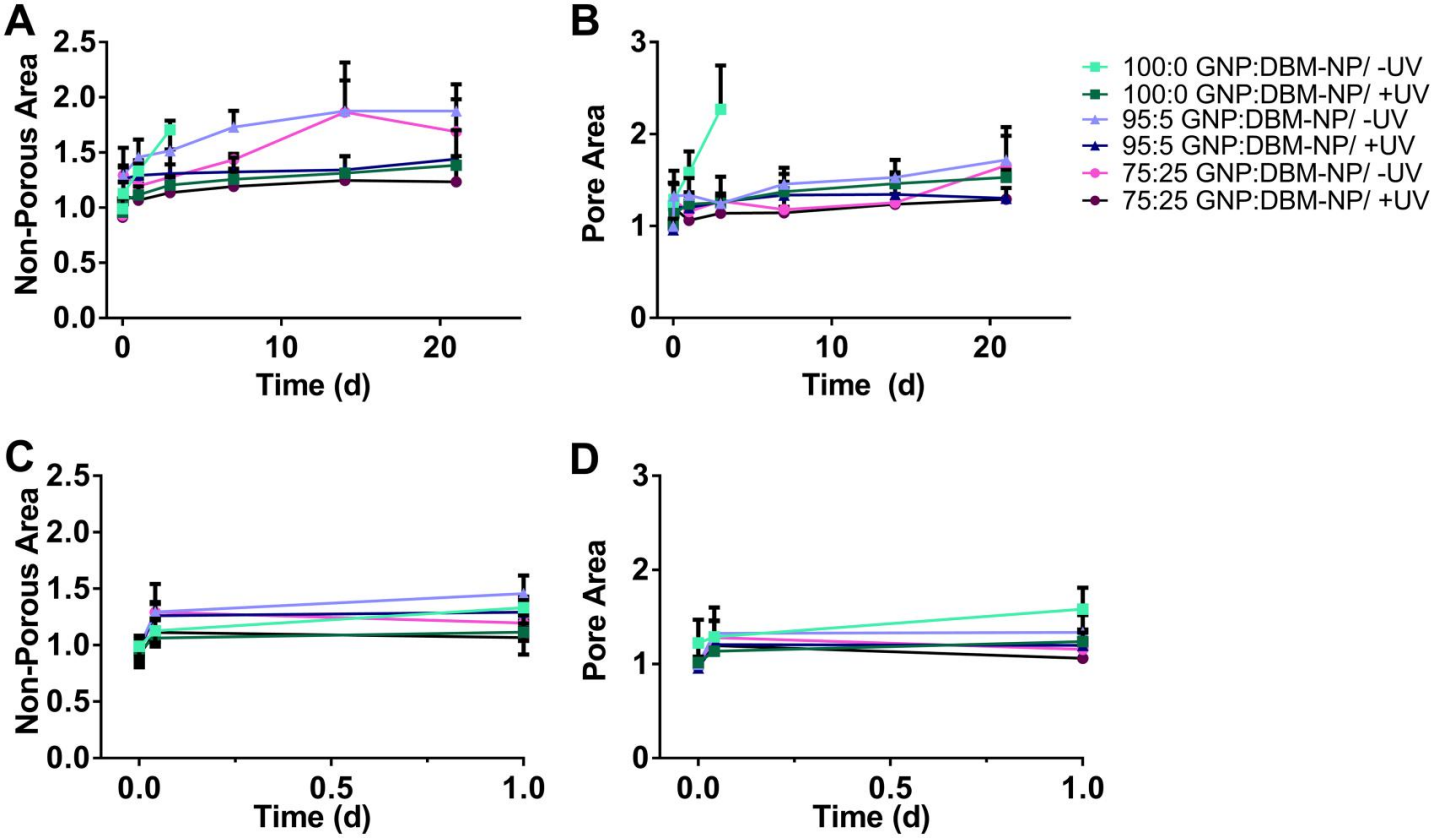
Quantified 3DP GNP:DBM-NP scaffold swelling in PBS as a function of DBM-NP-MA content and UV exposure for photocrosslinking. Changes in scaffold (**A**) non-porous area (area of hydrogel lattice) and (**B**) pore area (individual pore area averaged across all intact pores within scaffolds) over 21 days normalized to post-printing values for each scaffold. Expanded view of data presented in (A) and (B) for (**C**) non-porous area and (**D**) pore area over initial 24 h normalized to post-printing values for each scaffold. Error bars represent standard deviation (*n* = 3–4). Statistical significance is discussed in the Results section (*P* < 0.05).

3DP GNP:DBM-NP scaffolds were swollen over 21 days in PBS and the changes in scaffold dimensions relative to DBM-NP content and UV exposure were observed compared to initial post-printing features using image analysis. A schematic representation of the metrics assessed as scaffold non-porous area (hydrogel lattice area) and average pore area (individual pore area averaged across all intact pores within a scaffold) is found in Supplementary Fig. S3. As seen in Fig. 4, scaffolds without UV crosslinking showed more swelling over time compared to photocrosslinked scaffolds, particularly 100:0 GNP:DBM-NP scaffolds (Fig. 4A). Additionally, while uncrosslinked 95:5 (Fig. 4B) and 75:25 (Fig. 4C) GNP:DBM-NP scaffolds swelled over time, they did not lose their structural integrity over the 21-day period in PBS. These effects are quantified in Fig. 5 with scaffold and pore area for each scaffold normalized to post-printing dimensions with significant differences noted here (*P* < 0.05). Swelling over the first day, shown via changes in non-porous area (Fig. 5C) and average pore area (Fig. 5D), demonstrated significantly increased scaffold area for uncrosslinked 95:5 scaffolds compared to UV-crosslinked and uncrosslinked 75:25 GNP:DBM-NP scaffolds and UV-crosslinked 100:0 GNP:DBM-NP scaffolds. Additionally, uncrosslinked 100:0 GNP:DBM-NP had significantly increased scaffold area compared to UV-crosslinked 75:25 GNP:DBM-NP scaffolds. Pore area was significantly increased in uncrosslinked 100:0 GNP:DBM-NP scaffolds compared to all other scaffolds with the exception of uncrosslinked 95:5 GNP:DBM-NP scaffolds.

Additional effects of DBM-NP-MAs and UV crosslinking on swelling over the full 21 days are presented in Fig. 5A (scaffold non-porous area) and B (average pore area). Over the first 3 days, the 100:0 GNP:DBM-NP scaffolds swelled significantly, demonstrating a significantly higher increase in non-porous area compared to all but uncrosslinked 95:5 GNP:DBM-NP scaffolds and in average pore area compared to all groups. After this timepoint, the uncrosslinked 100:0 GNP:DBM-NP scaffolds no longer possessed the structural integrity for image analysis. Uncrosslinked 95:5 GNP:DBM-NP scaffolds showed significantly higher increases in scaffold non-porous area than all UV-crosslinked groups by Day 7. While uncrosslinked 75:25 GNP:DBM-NP scaffolds showed significantly higher non-porous area increases compared to all UV-crosslinked groups at Day 7, by Day 14, this difference was only significant when compared to UV-crosslinked 75:25 GNP:DBM-NP scaffolds. No other differences in average pore area among the remaining groups were noted until Day 14, at which time uncrosslinked 95:5 GNP:DBM-NP scaffolds were observed to have a significantly higher increase in average pore area than UV-crosslinked 95:5 and 75:25 GNP:DBM-NP scaffolds. Interestingly, these data indicate that uncrosslinked 75:25 GNP:DBM-NP scaffolds were not significantly different in swelling via average pore area compared to UV-crosslinked samples of other groups, showing that increased DBM-NP-MA content may have reduced swelling even in uncrosslinked samples.

### Degradation of 3DP GNP:DBM-NP scaffolds

**Figure 6.**
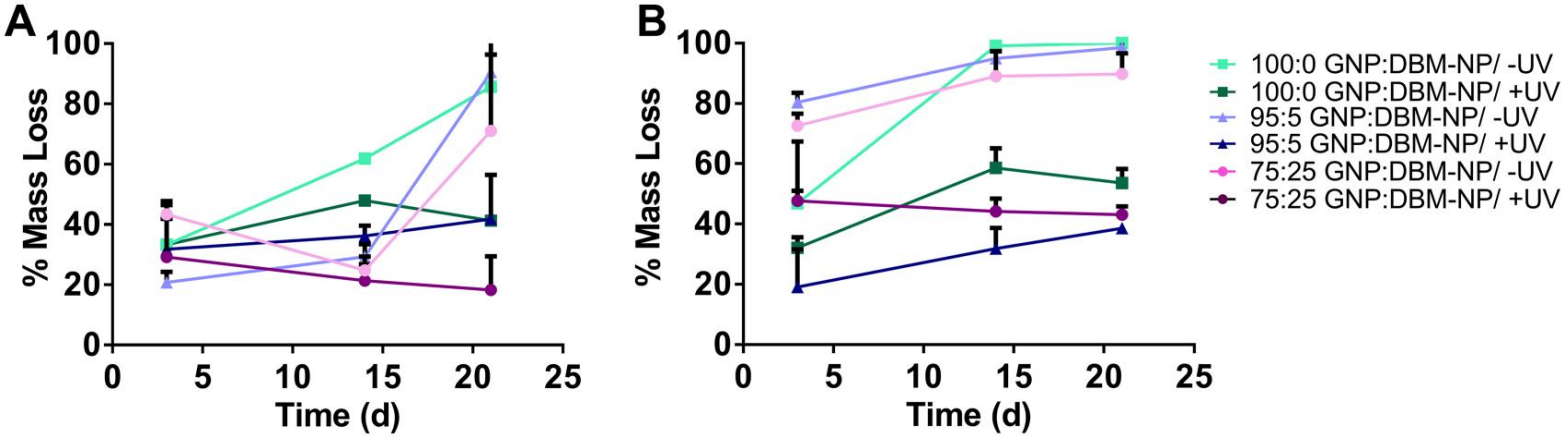
Quantified 3DP GNP:DBM-NP scaffold degradation over time relative to DBM-NP-MA content and UV exposure for photocrosslinking. Scaffold degradation over 21 days in (**A**) PBS, (**B**) PBS + 400 ng/ml collagenase. Error bars represent standard deviation (*n* = 3-4). Statistical significance is discussed in the Results section (*P* < 0.05).

In addition to swelling, the degradation of 3DP GNP:DBM-NP scaffolds in PBS and PBS + 400 ng/ml collagenase relative to DBM-NP content and UV exposure was evaluated over 21 days via changes in dry mass. Statistically significant differences are noted here (*P* < 0.05). In PBS (Fig. 6A), significant initial mass loss was observed for all groups at Day 3, akin to burst release, with the only significant difference observed between uncrosslinked 95:5 and 75:25 GNP:DBM-NP scaffolds. By Day 14, both uncrosslinked and UV-crosslinked 100:0 GNP:DBM-NP scaffolds saw significantly higher mass loss than both 75:25 GNP:DBM-NP groups, while only uncrosslinked 100:0 GNP-DBM-NP scaffolds had higher loss than 95:5 GNP:DBM-NP groups. At 21 days in PBS, all uncrosslinked groups showed significantly higher mass loss than UV-crosslinked groups and UV-crosslinked 75:25 scaffolds showed the least mass loss with little mass loss after the initial time point.

As shown in Fig. 6B, these observed effects are consistent with those observed for collagenase degradation. At Day 3, all uncrosslinked groups had significantly higher mass loss than all UV-crosslinked groups, and uncrosslinked 100:0 GNP:DBM-NP scaffolds showed significantly higher mass loss than the DBM-NP-MA-containing uncrosslinked groups. Significantly increased mass loss in uncrosslinked groups compared to UV-crosslinked groups remained through the 14- and 21-day time points. Among the UV-crosslinked groups, 95:5 GNP:DBM-NP scaffolds demonstrated significantly lower mass loss than 100:0 GNP:DBM-NP scaffolds but not 75:25 GNP:DBM-NP scaffolds through Days 14 and 21.

### In vitro *characterization of 3DP GNP:DBM-NP scaffold osteogenic potential*

**Figure 7.**
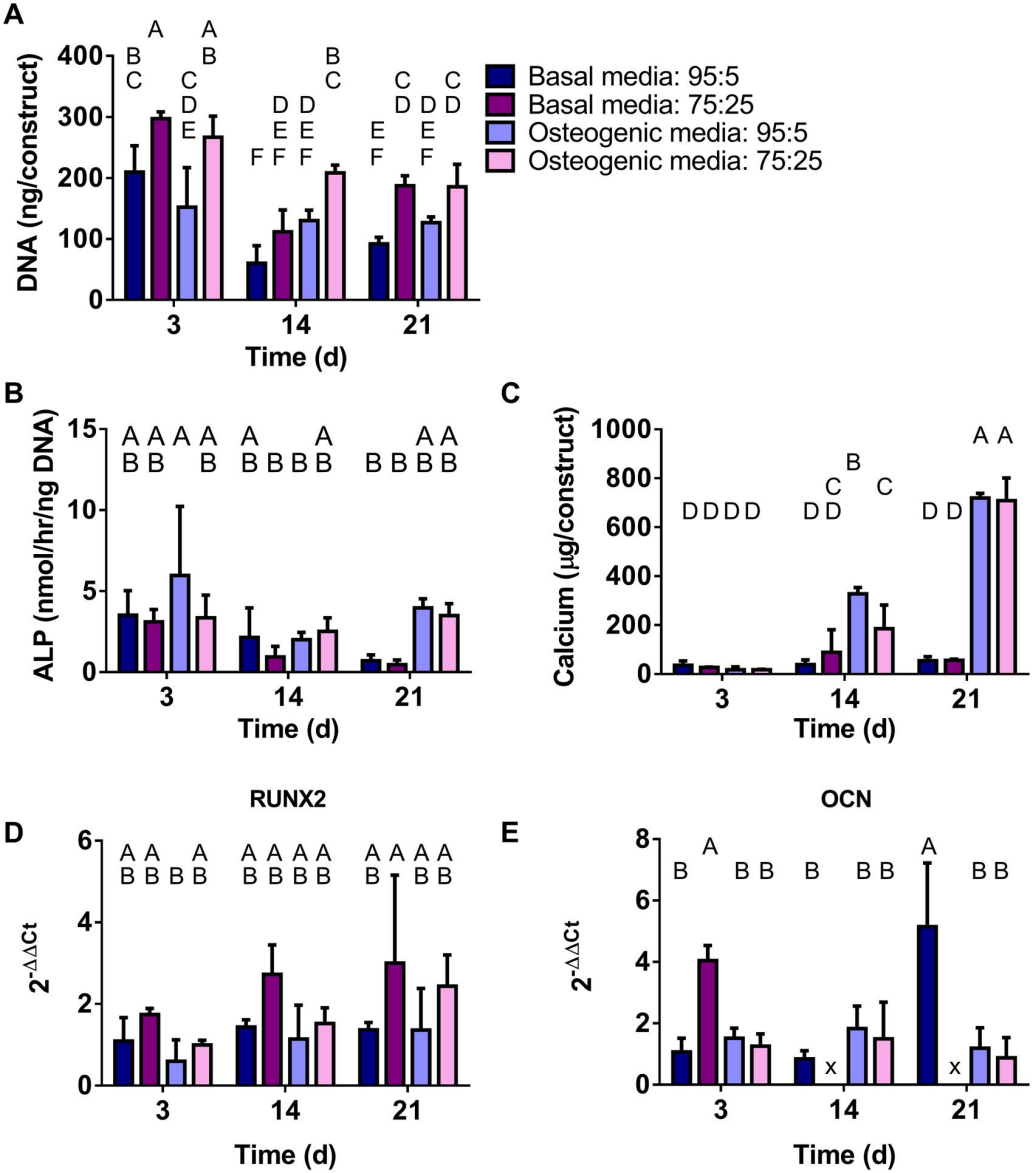
Evaluation of *in vitro* osteogenesis of DBM-NP-MA-incorporating scaffolds seeded with human bone marrow-derived mesenchymal stem cells relative to DBM-NP-MA content and culture media type. (**A**) DNA content quantified via PicoGreen assay. (**B**) ALP content quantified via ALP assay. (**C**) Calcium content quantified via calcium assay. qRT-PCR for relative (**D**) RUNX2 expression and (**E**) OCN expression. Shared letters indicate no significant difference between groups. × indicates no detectable gene expression at a given time point. Error bars represent standard deviation (*n* = 3–4, *P* < 0.05).

To investigate the osteogenic potential of DBM-NP-incorporating scaffolds, hbMSCs were seeded on low (95:5 GNP:DBM-NP) and high (75:25 GNP:DBM-NP) DBM-NP-MA content scaffolds and evaluated over 21 days for cell proliferation and differentiation. On PicoGreen analysis of DNA content, higher DBM-NP content scaffolds generally facilitated higher cell proliferation (Fig. 7A). On Day 3, 75:25 GNP:DBM-NP scaffolds in both basal and osteogenic media demonstrated higher DNA content than 95:5 GNP:DBM-NP scaffolds. 75:25 GNP:DBM-NP scaffolds in osteogenic media had the highest proliferation at Day 14, while other groups were similar. At Day 21, 75:25 GNP:DBM-NP basal and osteogenic media groups were similar, and the basal media group had higher DNA than that of its 95:5 GNP:DBM-NP counterpart. ALP activity was also assessed as an early to intermediate transient marker of osteogenesis (Fig. 7B). However, no clear effects emerged as all groups were similar at each time point, and the only differences observed were between 95:5 GNP:DBM-NP osteogenic media samples on Day 3 and the significantly lower on Day 14 75:25 GNP:DBM-NP basal media and 95:5 GNP:DBM-NP osteogenic media samples as well as basal media samples on Day 21. Next, calcium deposition was evaluated as a marker of late-stage osteogenic differentiation (Fig. 7C). Significant increases in calcium deposition were noted over time in osteogenic media groups for both 95:5 and 75:25 GNP:DBM-NP scaffolds. Day 14 samples from the 95:5 GNP:DBM-NP scaffolds in osteogenic media demonstrated higher calcium content than 75:25 GNP:DBM-NP scaffolds, but this difference was not observed for osteogenic media samples at the 21-day time point. Similar results were obtained with calcium content normalized to DNA content (Supplementary Fig. S4). Genetic expression of markers for both early- (RUNX2, Fig. 7D) and late- (OCN, Fig. 7E) stage osteogenesis was quantified with RT-qPCR. High variability in expression yielded no significant differences in RUNX2 expression within or across time points with the exception of 95:5 GNP:DBM-NP osteogenic media samples at Day 3 being significantly higher compared to 75:25 GNP:DBM-NP basal media samples at Day 21. OCN genetic expression also showed none of the hypothesized increases relative to DBM-NP content or media type. 75:25 GNP:DBM-NP basal media samples on Day 3 and 95:5 GNP:DBM-NP basal media samples on Day 21 had significantly higher OCN expression than other groups, while 75:25 GNP:DBM-NP scaffolds had no OCN expression on Days 14 and 21.

An additional smaller study comparing DNA content and calcium deposition on 100:0 GNP:DBM-NP and 0:100 GNP:DBM-NP scaffolds (see Supplementary Methods and Supplementary Fig. S5) showed similar results. No differences in DNA content, and therefore, cell proliferation, were observed after 3 days of culture (Supplementary Fig. S6). DBM-NP-MA scaffolds generally demonstrated increased DNA content compared to GNP-MA scaffolds at 14 and 21 days. DNA content for DBM-NP-MA scaffolds in basal media was similar to that in osteogenic media at both 14 and 21 days. At Day 14, GNP-MA basal and osteogenic media groups were similar to each other and lower than both DBM-NP-MA groups. GNP-MA scaffolds in basal media at 21 days had lower DNA content than all other groups while osteogenic media scaffolds had similar content to DBM-NP-MA groups. Calcium content (Supplementary Fig. S7) was similar between all groups on Day 3. However, at 14 days, the calcium content of osteogenic media groups was higher than those of basal media groups. DBM-NP-MA and GNP-MA osteogenic media groups showed similar mineralization per scaffold at 21 days, while calcium per ng DNA showed higher mineralization for GNP-MA osteogenic media scaffolds. Although basal media groups for both scaffold types were lower than osteogenic media groups, GNP-MA basal media scaffolds had significantly higher calcium deposition than DBM-NP-MA basal media scaffolds.

## Discussion

This study details the fabrication, characterization and application of DBM-NPs for 3DP aimed at bone regeneration. DBM has been widely clinically applied in bone augmentation and regeneration, including dental, craniofacial and spinal fusion applications [5, 6, 10]. As the non-mineral component of bone, DBM-derived materials provide inherent biochemical cues to mimic the native bone microenvironment, providing this innate bioactivity without the limited availability and donor site morbidity seen with autographs [6, 10]. In this study, bone was harvested from the legs of Sprague-Dawley SASCO rats and demineralized according to previously published protocols, and a two-step desolvation protocol modified from that used for GNPs was developed for the fabrication of nanoparticles from this DBM [22]. This fabrication required highly precise ratios of acetone to acetic acid and rates of acetone addition. Likewise, temperature proved a key component of the fabrication process, which was performed at 4°C to decrease the viscosity of the solubilized DBM and significantly increased the yield of DBM-NPs, in line with previously described collagen nanoparticle synthesis requiring low temperature [32].

Following methacryloylation of the nanoparticles, biochemical analysis of the DBM-NPs confirmed successful demineralization and decellularization with retention of collagen. The level of DNA present in DBM-NP-MAs was confirmed to be below the maximum acceptable level of 50 ng/mg for decellularization, above which clinical concern for an inflammatory reaction is increased [33]. Despite the purpose of decalcification, extreme removal of the calcium content has been shown to hamper osteoinductivity, since it also functions as providing nucleation site for remineralization in the future regeneration process [9]. An optimal range of 1–6% has been identified for demineralization, and the calcium content of the DBM-NP-MAs was found to be within the optimal range, confirming effective demineralization. Collagen content was similar between DBM and DBM-NP-MAs, indicating that nanoparticle synthesis did not impact the collagen content of the DBM. The significant difference between the sGAG contents of DBM and DBM-NP-MAs provides evidence that further processing decreases the sGAG content in the material. The concentration of the methacryloyl groups for both DBM-NP-MAs and GNP-MAs was quantified using a TMSP standard, as previously published [34]. GNP-MAs were demonstrated to be more highly functionalized with methacryloyl groups by mass than DBM-NP-MAs. Additionally, these quantitative analyses demonstrate that methacrylic anhydride reacted most with free amine groups on the nanoparticles, as previously reported [22, 35]. These results confirm that DBM-NP-MAs contain key biochemical components of DBM and that they are functionalized for photocrosslinking.

DBM-NP-MAs were then incorporated into colloidal composite inks in varying ratios with GNP-MAs. A previous study demonstrated the ability to create stable, photoreactive extrusion 3DP inks using GNP-MAs [22]. Here, DBM-NP-MAs were introduced to colloidal inks to provide specific biochemical cues for bone regeneration not provided by gelatin alone. As shown in Fig. 2, inks incorporating DBM-NP-MAs at both lower and higher concentrations (95:5 GNP:DBM-NP and 75:25 GNP:DBM-NP, with both nanoparticle types methacryloylated) did not significantly impact rheological characteristics associated with printability, including shear-thinning behavior and recovery of gel-like behavior following high deformation in the form of a strain ramp. Rapid recovery of *G*′ and *G*″ following high shear replicates how these properties will adapt following extrusion, crucial for maintaining the shape fidelity of the printed fibers [22, 36, 37]. This thixotropic behavior has been previously observed for colloidal composite systems, as with collagen and hyaluronic acid inks supplemented with GNPs for increased printability, and relies on the outsized impact that relatively weak interactions, such as van der Waals forces and electrostatic interactions, have on the nanoscale particles with necessarily high surface area-to-volume ratios [22, 38]. The frequency-independent behavior of these GNP:DBM-NP inks shows rapid reformation of these noncovalent bonds. These interactions and their rapid recovery with return to a low-shear environment enable colloidal systems to recover their elastic properties following extrusion [38]. In addition, the yield stress and strain are similar between all inks, which is important because low yield stress and strain can lead to fiber spreading or collapse after deposition. A yield stress of at least 100 Pa has been recommended for high print fidelity, which values for GNP:DBM-NP inks exceed for all formulations [39]. The similarities in rapid recovery behavior and yield stress and strain between inks of all DBM-NP-MA concentrations demonstrate that these nanoparticles are capable of forming such a stable colloidal network with GNP-MAs. The results of this rheological analysis mirror those for GNP-MA-only inks as previously published, with similarities in both obtained values and observed properties [22]. The retention of these properties is key in maintaining fiber shape fidelity and overall post-extrusion stability prior to UV crosslinking, which were demonstrated in images of printed scaffolds (Fig. 3D).

Likewise, DBM-NP-MA incorporation into GNP-MA inks did not reduce the photoreactivity of the colloidal composite inks. Methacryloylation has been widely explored for bioinks including decellularized ECMs as a means of rapid crosslinking with UV exposure and has been shown to have no significant impact on ECM-based material bioactivity [40–45]. NMR data (Fig. 1E and F) confirm the successful methacryloylation of DBM-NPs and GNPs, and, interestingly, integration of methacrylate and methacrylamide peaks showed a lower molar concentration of methacryloyl groups for DBM-NPs compared to GNP-MAs. However, Fig. 3 shows that all inks displayed significant increases in storage and loss moduli with UV exposure. This result indicates that a minimum threshold of methacryloyl groups was present to achieve the formation of a reinforcing covalent inter-particle network. Additionally, after UV crosslinking, the morphology of printed scaffolds remained stable.

The stability of these networks was further physicochemically evaluated relative to DBM-NP-MA content and UV crosslinking with 3DP scaffold swelling and degradation. Of note, uncrosslinked GNP-MA scaffolds were not stable in PBS over the full 21 days of the study, with complete scaffold dissolution occurring between 3 and 7 days. In contrast, both low and high DBM-NP-MA-incorporating uncrosslinked scaffolds persisted throughout the entire length of the swelling study. This rapid degradation of uncrosslinked GNP-MA scaffolds has been previously attributed to the partial degradation of glutaraldehyde-based covalent intraparticle crosslinking by the acidic environment generated by the byproduct methacrylic acid during the methacryloylation reaction [22]. The results observed here suggest that the addition of DBM-NP-MAs serves to further stabilize the noncovalent interparticle network within these inks. Because DBM-NP-MAs are larger, GNP-MAs may serve as a filler between them, increasing the packing density of the composite and decreasing the distance of noncovalent bonds, as component size is a key consideration in composite nanoparticle materials [46]. Additionally, DBM-NP-MAs contain significant amounts of collagen, which contains hydrophobic domains with stable tertiary structure, unlike gelatin [47]. This property, as well as the complex non-collagen protein component of DBM, may lead to increased inter-particle hydrophobic and electrostatic interactions in an aqueous environment. Over the course of 21 days, uncrosslinked scaffolds of all GNP:DBM-NP ratios showed significant swelling in terms of scaffold area compared to their UV-crosslinked scaffolds. This difference illustrates the utility of generating a covalent network within these 3DP scaffolds and the ability to tune scaffold swelling using photoreactive moieties. In addition, average pore area was increased for uncrosslinked 100:0 and 95:5 GNP:DBM-NP scaffolds compared to UV-crosslinked scaffolds, indicating that even with scaffold swelling, open pores were maintained. The maintenance of designed pores is essential in bone tissue engineering, facilitating nutrient and waste transport as well as vascular ingrowth [48]. This colloidal composite system, therefore, enables precise design for 3DP scaffold swelling without the loss of essential porosity.

The degradation of these scaffolds followed a similar pattern. In PBS, by the end of 21 days, UV-crosslinked scaffolds proved to have significantly less mass loss than uncrosslinked groups. Interestingly, UV-crosslinked 75:25 GNP:DBM-NP scaffolds also demonstrated significantly less mass loss compared to 100:0 GNP:DBM-NP scaffolds. In collagenase, again, uncrosslinked scaffolds demonstrated increased mass loss compared to UV-crosslinked scaffolds. In this case, the UV-crosslinked 95:5 GNP:DBM-NP scaffolds demonstrated less mass loss compared to UV-crosslinked 100:0 GNP:DBM-NP scaffolds. The persistence of these DBM-NP-MA groups again demonstrates that the incorporation of DBM-NP-MAs, although seen here in varying amounts, may increase the aqueous stability of 3DP colloidal composite scaffolds and reinforce the UV-generated covalent interparticle network. This study also demonstrates the potential for controlled degradation of ECM-incorporating materials in regenerative medicine applications, allowing for temporal precision in biochemical cue presentation and release.

The osteoconductivity and osteoinductivity of low and high DBM-NP-MA-content scaffolds were then evaluated. Increased DBM-NP content supported increased cell proliferation at earlier timepoints in both media types, with the 75:25 GNP:DBM-NP group having higher DNA values than 95:5 GNP:DBM-NP scaffolds at 21 days in basal media. This difference in proliferation may be attributed to the trophic factors found within the DBM-NP-MAs that increase their bioactivity compared to gelatin alone [5, 6, 10]. When looking at ALP activity, no clear effects emerged over the three assayed timepoints. However, calcium content increased over time in both groups in osteogenic media. ALP is a transient early marker of osteogenesis and has been found to have little correlation to calcium deposition and late-stage osteogenic markers [49, 50]. Likewise, RUNX2 and OCN gene expression, early- and late-stage markers of osteogenesis, respectively, did not show clear differences across groups and time points. The mineralization of DBM-NP-MA-incorporating scaffolds in osteogenic media demonstrates the osteoconductive properties of these scaffolds. However, the lack of calcium deposition for basal media groups does not support osteoinductivity. Likewise, DBM osteoinductivity in the setting of MSC osteogenesis has been previously reported for scaffolds largely or entirely composed of DBM [51–53]. However, the universality of this osteoinductivity for DBM-derived materials has been questioned based on the complex composition of DBM, which may contain bioactive molecules that downregulate osteogenesis [54, 55]. For instance, it was demonstrated that dermapontin, identified as one of the most abundant low molecular weight proteins in DBM, has an inhibitory effect on BMP activity in osteogenic precursor cells [56]. The downregulation of mineralization in the DBM-NP-MA-only basal media group compared to the GNP-MA-only basal media group supports the idea of inhibitory factors stalling calcium deposition by MSCs on DBM-NPs. This explanation also sheds light on the lack of OCN expression in the high DBM-NP-MA content group (75:25 GNP:DBM-NP) in basal media compared to low but measurable expression in the 95:5 GNP:DBM-NP group. Alternatively, these results could suggest that growth factors, such as BMPs, associated with the osteoinductivity of DBM were denatured or lost throughout the DBM-NP-MA synthesis and methacryloylation processing steps. Likewise, hydrogel stiffness, demonstrated with collagen hydrogels, has been shown to play a significant role in upregulating MSC osteogenesis [57]. In pure DBM-NP-MA and GNP-MA scaffolds, the higher methacryloyl group concentration of GNP-MAs compared to DBM-NP-MAs may have resulted in decreased DBM-NP-MA scaffold stiffness after photocrosslinking compared to GNP-MA scaffolds, which may, in turn, have resulted in decreased mechanical cues for MSC osteogenic differentiation for DBM-NP-MA scaffolds.

Partially DBM has also been shown to significantly increase osteogenic differentiation of bone marrow-derived stem cells, indicating that the provision of the mineral component within these colloidal composite systems might improve osteoinductivity [58]. Recent studies combining DBM with nano-hydroxyapatite saw increased evidence of *in vitro* human MSC osteogenesis and *in vivo* bone formation, suggesting that the addition of this nanomaterial and its mineral cues may be beneficial within a colloidal composite system [19, 59]. Likewise, additional osteogenic proteins such as osteogenic protein-1 have been shown to increase the *in vitro* osteoinductivity of DBM for human MSCs [60]. The DBM-NP and GNP colloidal platform could also serve as a reservoir for supplementary growth factor or drug delivery with controlled release from the scaffold bulk tuned via glutaraldehyde intraparticle crosslinking and UV-mediated interparticle crosslinking.

The data here suggest that novel ECM-based nanoparticles may be fabricated and integrated into colloidal composite systems without significantly impacting properties such as viscosity and recovery behavior that determine extrudability and 3DP print fidelity. Additionally, the functionalization of these colloidal building blocks enables photocrosslinking. This study demonstrates that this process results in control over hydrogel swelling and degradation. These results show the potential for the development of ECM-based nanoparticles to expand to a broad range of tissues. ECM-based nanoparticles could then be incorporated within colloidal composite systems for 3DP or within injectable platforms. The generation of photoreactive ECM-based nanoparticles for 3DP in composite inks, therefore, represents a versatile tool for regenerative medicine, offering a system for controlled swelling and degradation as well as the modular introduction of additional materials or biomolecules.

## Conclusions

In this study, novel photoreactive DBM-NP-MAs were developed and introduced into colloidal composite 3DP inks in different ratios with GNP-MAs (100:0, 95:5 and 75:25 GNP:DBM-NP). These inks demonstrated high printability with no disruption in the colloidal noncovalent network upon incorporation of DBM-NP-MAs demonstrated through the retention of shear-thinning and recovery behavior. Additionally, due to the photocrosslinkable nature of the colloidal components, physicochemical properties such as scaffold swelling and degradation were controlled with UV crosslinking. Uncrosslinked scaffolds for all DBM-NP-MA content levels demonstrated increased changes in scaffold area and average pore size compared to UV-crosslinked scaffolds. Likewise, significantly less mass loss was observed over 21 days in both PBS and collagenase for scaffolds that were photocrosslinked. Uncrosslinked DBM-NP-MA-incorporating scaffolds demonstrated greater stability while swelling than 100:0 GNP:DBM-NP scaffolds, thought to be the result of additional noncovalent interactions with the complexity of DBM composition and DBM-NP-MA size. In an *in vitro* osteogenic study with bone marrow-derived MSCs, both 95:5 and 75:25 GNP:DBM-NP scaffolds showed similar osteoconductivity, demonstrated by calcium deposition in osteogenic media. These photocrosslinkable DBM-NP-MA and GNP-MA colloidal composite inks demonstrate a platform for developing ECM-derived colloidal materials and controlling the temporal presentation of biochemical cues for tissue engineering.

## Supplementary Material

rbad090_Supplementary_Data

## References

[1] Ashman A. Ridge preservation: important buzzwords in dentistry. Gen Dent 2000;48:304–12. 11199597

[2] Louis PJ , Gutta R, Said-Al-Naief N, Bartolucci AA. Rescaffoldion of the maxilla and mandible with particulate bone graft and titanium mesh for implant placement. J Oral Maxillofac Surg 2008;66:235–45. 18201602 10.1016/j.joms.2007.08.022

[3] Salyer KE , Taylor DP. Bone grafts in craniofacial surgery. Clin Plast Surg 1987;14:27–35. 3545620

[4] Fiorellini JP , Nevins ML. Localized ridge augmentation/preservation. A systematic review. Ann Periodontol 2003;8:321–7. 14971259 10.1902/annals.2003.8.1.321

[5] Shepard NA , Rush AJ, Scarborough NL, Carter AJ, Phillips FM. Demineralized bone matrix in spine surgery: a review of current applications and future trends. Int J Spine Surg 2021;15:113–9. 10.14444/8059 PMC8092935 34376500

[6] Zhang H , Yang L, Yang X, Wang F, Feng J, Hua K, Li Q, Hu Y. Demineralized bone matrix carriers and their clinical applications: an overview. Orthop Surg 2019;11:725–37. 31496049 10.1111/os.12509 PMC6819172

[7] Pati F , Jang J, Ha D-H, Won Kim S, Rhie J-W, Shim J-H, Kim D-H, Cho D-W. Printing three-dimensional tissue analogues with decellularized extracellular matrix bioink. Nat Commun 2014;5:3935. 24887553 10.1038/ncomms4935 PMC4059935

[8] Bedell ML , Torres AL, Hogan KJ, Wang Z, Wang B, Melchiorri AJ, Grande-Allen KJ, Mikos AG. Human gelatin-based composite hydrogels for osteochondral tissue engineering and their adaptation into bioinks for extrusion, inkjet, and digital light processing bioprinting. Biofabrication 2022;14:45012. 10.1088/1758-5090/ac8768 PMC9633045 35931060

[9] Zhang M , Powers RM Jr, Wolfinbarger L Jr. Effect(s) of the demineralization process on the osteoinductivity of demineralized bone matrix. J Periodontol 1997;68:1085–92. 9407401 10.1902/jop.1997.68.11.1085

[10] Gruskin E , Doll BA, Futrell FW, Schmitz JP, Hollinger JO. Demineralized bone matrix in bone repair: history and use. Adv Drug Deliv Rev 2012;64:1063–77. 22728914 10.1016/j.addr.2012.06.008 PMC7103314

[11] Ramis JM , Blasco‐Ferrer M, Calvo J, Villa O, Cladera MM, Corbillo C, Gayà A, Monjo M. Improved physical and osteoinductive properties of demineralized bone matrix by gelatin methacryloyl formulation. J Tissue Eng Regen Med 2020;14:475–85. 32011080 10.1002/term.3012

[12] Roberts TT , Rosenbaum AJ. Bone grafts, bone substitutes and orthobiologics. Organogenesis 2012;8:114–24. 23247591 10.4161/org.23306 PMC3562252

[13] Kang H-J , Park S-S, Tripathi G, Lee B-T. Injectable demineralized bone matrix particles and their hydrogel bone grafts loaded with β-tricalcium phosphate powder and granules: a comparative study. Mater Today Bio 2022;16:100422. 10.1016/j.mtbio.2022.100422 PMC9483747 36133794

[14] Parthiban SP , Athirasala A, Tahayeri A, Abdelmoniem R, George A, Bertassoni LE. BoneMA—synthesis and characterization of a methacrylated bone-derived hydrogel for bioprinting of in-vitro vascularized tissue constructs. Biofabrication 2021;13:35031. 10.1088/1758-5090/abb11f PMC9059555 35130535

[15] Li D , Yang Z, Zhao X, Luo Y, Ou Y, Kang P, Tian M. A bone regeneration strategy via dual delivery of demineralized bone matrix powder and hypoxia-pretreated bone marrow stromal cells using an injectable self-healing hydrogel. J Mater Chem B 2021;9:479–93. 33289774 10.1039/d0tb01924k

[16] Zhao Y , Lin H, Zhang J, Chen B, Sun W, Wang X, Zhao W, Xiao Z, Dai J. Crosslinked three-dimensional demineralized bone matrix for the adipose-derived stromal cell proliferation and differentiation. Tissue Eng Part A 2009;15:13–21. 18652539 10.1089/ten.tea.2008.0039

[17] Leszczak V , Place LW, Franz N, Popat KC, Kipper MJ. Nanostructured biomaterials from electrospun demineralized bone matrix: a survey of processing and crosslinking strategies. ACS Appl Mater Interfaces 2014;6:9328–37. 24865253 10.1021/am501700e

[18] Lin H , Zhao Y, Sun W, Chen B, Zhang J, Zhao W, Xiao Z, Dai J. The effect of crosslinking heparin to demineralized bone matrix on mechanical strength and specific binding to human bone morphogenetic protein-2. Biomaterials 2008;29:1189–97. 18083224 10.1016/j.biomaterials.2007.11.032

[19] Plantz MA , Minardi S, Lyons JG, Greene AC, Ellenbogen DJ, Hallman M, Yamaguchi JT, Jeong S, Yun C, Jakus AE, Blank KR, Havey RM, Muriuki M, Patwardhan AG, Shah RN, Hsu WK, Stock SR, Hsu EL. Osteoinductivity and biomechanical assessment of a 3D printed demineralized bone matrix-ceramic composite in a rat spine fusion model. Acta Biomater 2021;127:146–58. 33831576 10.1016/j.actbio.2021.03.060 PMC8154748

[20] Driscoll JA , Lubbe R, Jakus AE, Chang K, Haleem M, Yun C, Singh G, Schneider AD, Katchko KM, Soriano C, Newton M, Maerz T, Li X, Baker K, Hsu WK, Shah RN, Stock SR, Hsu EL. 3D-printed ceramic-demineralized bone matrix hyperelastic bone composite scaffolds for spinal fusion. Tissue Eng Part A 2020;26:157–66. 31469055 10.1089/ten.tea.2019.0166 PMC7044791

[21] Plantz M , Lyons J, Yamaguchi JT, Greene AC, Ellenbogen DJ, Hallman MJ, Shah V, Yun C, Jakus AE, Procissi D, Minardi S, Shah RN, Hsu WK, Hsu EL. Preclinical safety of a 3D-printed hydroxyapatite-demineralized bone matrix scaffold for spinal fusion. Spine (Phila Pa 1976) 2022;47:82–9. 34115714 10.1097/BRS.0000000000004142 PMC8765284

[22] Diba M , Koons GL, Bedell ML, Mikos AG. 3D printed colloidal biomaterials based on photo-reactive gelatin nanoparticles. Biomaterials 2021;274:120871. 34029914 10.1016/j.biomaterials.2021.120871 PMC8196631

[23] Kinard LA , Dahlin RL, Lam J, Lu S, Lee EJ, Kasper FK, Mikos AG. Synthetic biodegradable hydrogel delivery of demineralized bone matrix for bone augmentation in a rat model. Acta Biomater 2014;10:4574–82. 25046637 10.1016/j.actbio.2014.07.011 PMC4186894

[24] Chen I-C , Su C-Y, Lai C-C, Tsou Y-S, Zheng Y, Fang H-W. Preparation and characterization of moldable demineralized bone matrix/calcium sulfate composite bone graft materials. J Funct Biomater 2021;12:56. 34698233 10.3390/jfb12040056 PMC8544512

[25] Parmaksiz M , Lalegül-Ülker Ö, Vurat MT, Elçin AE, Elçin YM. Magneto-sensitive decellularized bone matrix with or without low frequency-pulsed electromagnetic field exposure for the healing of a critical-size bone defect. Mater Sci Eng C Mater Biol Appl 2021;124:112065. 33947558 10.1016/j.msec.2021.112065

[26] Cissell DD , Link JM, Hu JC, Athanasiou KA. A modified hydroxyproline assay based on hydrochloric acid in ehrlich’s solution accurately measures tissue collagen content. Tissue Eng Part C Methods 2017;23:243–50. 28406755 10.1089/ten.tec.2017.0018 PMC5397204

[27] Zheng CH , Levenston ME. Fact versus artifact: avoiding erroneous estimates of sulfated glycosaminoglycan content using the dimethylmethylene blue colorimetric assay for tissue-engineered constructs. Eur Cell Mater 2015;29:224–36; discussion 236. 25890595 10.22203/ecm.v029a17 PMC4445729

[28] Zhu M , Wang Y, Ferracci G, Zheng J, Cho N-J, Lee BH. Gelatin methacryloyl and its hydrogels with an exceptional degree of controllability and batch-to-batch consistency. Sci Rep 2019;9:6863. 31053756 10.1038/s41598-019-42186-x PMC6499775

[29] Yoshihara Y , Nakamura H, Obata K, Yamada H, Hayakawa T, Fujikawa K, Okada Y. Matrix metalloproteinases and tissue inhibitors of metalloproteinases in synovial fluids from patients with rheumatoid arthritis or osteoarthritis. Ann Rheum Dis 2000;59:455–61. 10834863 10.1136/ard.59.6.455 PMC1753174

[30] Bedell ML , Wang Z, Hogan KJ, Torres AL, Pearce HA, Chim LK, Grande-Allen KJ, Mikos AG. The effect of multi-material architecture on the ex vivo osteochondral integration of bioprinted constructs. Acta Biomater 2023;155:99–112. 36384222 10.1016/j.actbio.2022.11.014 PMC9805529

[31] Smith BT , Bittner SM, Watson E, Smoak MM, Diaz-Gomez L, Molina ER, Kim YS, Hudgins CD, Melchiorri AJ, Scott DW, Grande-Allen KJ, Yoo JJ, Atala A, Fisher JP, Mikos AG. Multimaterial dual gradient three-dimensional printing for osteogenic differentiation and spatial segregation. Tissue Eng Part A 2020;26:239–52. 31696784 10.1089/ten.tea.2019.0204 PMC7133451

[32] Rathore P , Arora I, Rastogi S, Akhtar M, Singh S, Samim M. Collagen nanoparticle-mediated brain silymarin delivery: an approach for treating cerebral ischemia and reperfusion-induced brain injury. Front Neurosci 2020;14:538404. 33192240 10.3389/fnins.2020.538404 PMC7649428

[33] Crapo PM , Gilbert TW, Badylak SF. An overview of tissue and whole organ decellularization processes. Biomaterials 2011;32:3233–43. 21296410 10.1016/j.biomaterials.2011.01.057 PMC3084613

[34] Claaßen C , Claaßen MH, Truffault V, Sewald L, Tovar GEM, Borchers K, Southan A. Quantification of substitution of gelatin methacryloyl: best practice and current pitfalls. Biomacromolecules 2018;19:42–52. 29211461 10.1021/acs.biomac.7b01221

[35] Shirahama H , Lee BH, Tan LP, Cho N-J. Precise tuning of facile One-Pot gelatin methacryloyl (GelMA) synthesis. Sci Rep 2016;6:31036. 27503340 10.1038/srep31036 PMC4977492

[36] Jain T , Baker HB, Gipsov A, Fisher JP, Joy A, Kaplan DS, Isayeva I. Impact of cell density on the bioprinting of gelatin methacrylate (GelMA) bioinks. Bioprinting 2021;22:e00131.

[37] Clark CC , Aleman J, Mutkus L, Skardal A. A mechanically robust thixotropic collagen and hyaluronic acid bioink supplemented with gelatin nanoparticles. Bioprinting 2019;16:e00058.

[38] Zhu C , Pascall AJ, Dudukovic N, Worsley MA, Kuntz JD, Duoss EB, Spadaccini CM. Colloidal materials for 3D printing. Annu Rev Chem Biomol Eng 2019;10:17–42. 30951639 10.1146/annurev-chembioeng-060718-030133

[39] Townsend JM , Beck EC, Gehrke SH, Berkland CJ, Detamore MS. Flow behavior prior to crosslinking: the need for precursor rheology for placement of hydrogels in medical applications and for 3D bioprinting. Prog Polym Sci 2019;91:126–40. 31571701 10.1016/j.progpolymsci.2019.01.003 PMC6768569

[40] Lee J , Hong J, Kim W, Kim GH. Bone-derived dECM/alginate bioink for fabricating a 3D cell-laden mesh structure for bone tissue engineering. Carbohydr Polym 2020;250:116914. 33049834 10.1016/j.carbpol.2020.116914

[41] Elomaa L , Keshi E, Sauer IM, Weinhart M. Development of GelMA/PCL and dECM/PCL resins for 3D printing of acellular in vitro tissue scaffolds by stereolithography. Mater Sci Eng C Mater Biol Appl 2020;112:110958. 32409091 10.1016/j.msec.2020.110958

[42] Kim W , Lee H, Lee J, Atala A, Yoo JJ, Lee SJ, Kim GH. Efficient myotube formation in 3D bioprinted tissue construct by biochemical and topographical cues. Biomaterials 2020;230:119632. 31761486 10.1016/j.biomaterials.2019.119632 PMC7141931

[43] Lee H , Kim W, Lee J, Yoo JJ, Kim GH, Lee SJ. Effect of hierarchical scaffold consisting of aligned dECM nanofibers and poly (lactide-co-glycolide) struts on the orientation and maturation of human muscle progenitor cells. ACS Appl Mater Interfaces 2019;11:39449–58. 31584255 10.1021/acsami.9b12639

[44] Jung M , Han Y, Woo C, Ki CS. Pulmonary tissue-mimetic hydrogel niches for small cell lung cancer cell culture. J Mater Chem B 2021;9:1858–66. 33533364 10.1039/d0tb02609c

[45] Kim W , Lee H, Lee CK, Kyung JW, An SB, Han I, Kim GH. A bioprinting process supplemented with in situ electrical stimulation directly induces significant myotube formation and myogenesis. Adv Funct Mater 2021;31:2105170.

[46] Diba M , Spaans S, Ning K, Ippel BD, Yang F, Loomans B, Dankers PYW, Leeuwenburgh SCG. Self-healing biomaterials: from molecular concepts to clinical applications. Adv Mater Interfaces 2018;5:1800118.

[47] Shoulders MD , Raines RT. Collagen structure and stability. Annu Rev Biochem 2009;78:929–58. 19344236 10.1146/annurev.biochem.77.032207.120833 PMC2846778

[48] Koons GL , Diba M, Mikos AG. Materials design for bone-tissue engineering. Nat Rev Mater 2020;5:584–603.

[49] Souza TFB , Sakamoto SS, Ferreira GTNM, Gameiro R, Marinho M, de Andrade AL, Cardoso TC. Osteogenic potential of mesenchymal cells derived from canine umbilical cord matrix co-cultured with platelet-rich plasma and demineralized bone matrix. J Vet Sci 2015;16:381–4. 26040617 10.4142/jvs.2015.16.3.381 PMC4588025

[50] Hoemann CD , El-Gabalawy H, McKee MD. In vitro osteogenesis assays: influence of the primary cell source on alkaline phosphatase activity and mineralization. Pathol Biol (Paris) 2009;57:318–23. 18842361 10.1016/j.patbio.2008.06.004

[51] Kang E-J , Byun J-H, Choi Y-J, Maeng G-H, Lee S-L, Kang D-H, Lee J-S, Rho G-J, Park B-W. In vitro and in vivo osteogenesis of porcine skin-derived mesenchymal stem cell-like cells with a demineralized bone and fibrin glue scaffold. Tissue Eng Part A 2010;16:815–27. 19778183 10.1089/ten.TEA.2009.0439

[52] Park B-W , Kang E-J, Byun J-H, Son M-G, Kim H-J, Hah Y-S, Kim T-H, Mohana Kumar B, Ock S-A, Rho G-J. In vitro and in vivo osteogenesis of human mesenchymal stem cells derived from skin, bone marrow and dental follicle tissues. Differentiation 2012;83:249–59. 22469856 10.1016/j.diff.2012.02.008

[53] Mattioli-Belmonte M , Montemurro F, Licini C, Iezzi I, Dicarlo M, Cerqueni G, Coro F, Vozzi G. Cell-free demineralized bone matrix for mesenchymal stem cells survival and colonization. Materials (Basel) 2019;12:1360. doi: 10.3390/ma12091360. PMC6538993 31027339

[54] Behnam K , Brochmann EJ, Murray SS. Alkali-urea extraction of demineralized bone matrix removes noggin, an inhibitor of bone morphogenetic proteins. Connect Tissue Res 2004;45:257–60. 15763935 10.1080/03008200490903048

[55] Chen C , Rehnama M, Kim S, Lee C-S, Zhang X, Aghaloo T, Fan J, Lee M. Enhanced osteoinductivity of demineralized bone matrix with noggin suppression in polymer matrix. Adv Biol 2021;5:2000135. 10.1002/adbi.202000135 PMC7877805 33585837

[56] Behnam K , Murray SS, Brochmann EJ. BMP stimulation of alkaline phosphatase activity in pluripotent mouse C2C12 cells is inhibited by dermatopontin, one of the most abundant low molecular weight proteins in demineralized bone matrix. Connect Tissue Res 2006;47:271–7. 17118749 10.1080/03008200600995908

[57] Kelly DJ , Jacobs CR. The role of mechanical signals in regulating chondrogenesis and osteogenesis of mesenchymal stem cells. Birth Defects Res C Embryo Today 2010;90:75–85. 20301221 10.1002/bdrc.20173

[58] Liu S , Wang Y, Wang J, Qiu P, Wang S, Shi Y, Li M, Chen P, Lin X, Fang X. A cancellous bone matrix system with specific mineralisation degrees for mesenchymal stem cell differentiation and bone regeneration. Biomater Sci 2019;7:2452–67. 30942200 10.1039/c8bm01657g

[59] Nicoletti A , Torricelli P, Bigi A, Fornasari P, Fini M, Moroni L. Incorporation of nanostructured hydroxyapatite and poly(N-isopropylacrylamide) in demineralized bone matrix enhances osteoblast and human mesenchymal stem cell activity. Biointerphases 2015;10:041001. 26443012 10.1116/1.4931882

[60] Tsiridis E , Ali Z, Bhalla A, Gamie Z, Heliotis M, Gurav N, Deb S, DiSilvio L. In vitro proliferation and differentiation of human mesenchymal stem cells on hydroxyapatite versus human demineralised bone matrix with and without osteogenic protein-1. Expert Opin Biol Ther 2009;9:9–19. 19063689 10.1517/14712590802622473

