# Supplementary material for: Development of photoreactive demineralized bone matrix 3D printing colloidal inks for bone tissue engineering
Source: Regen Biomater. 2023 Oct 19;10:rbad090. doi: 10.1093/rb/rbad090 (PMC10634525; doi:10.1093/rb/rbad090)
Supplement: rbad090_Supplementary_Data [file rbad090_supplementary_data.docx]

**Development of Photoreactive Demineralized Bone Matrix 3D-Printing Colloidal Inks for Bone Tissue Engineering**

Katie J. Hogan, Hayriye Öztatlı, Marissa Perez, Sophia Si, Reyhan Umurhan, Elysa Jui, Ziwen Wang, Emily Jiang, Sa Han, Mani Diba, K. Jane Grande-Allen, Bora Garipcan, Antonios G. Mikos

**Supplementary Methods**

*S1.1 Preparation of GNP-MA and DBM-NP-MA colloidal inks*

GNP-MA and DBM-NP-MA 3DP inks were both made using 20 w/v% nanoparticle content. A concentration of 0.5 w/v% Irgacure 2959 was dissolved in PBS, which was then added to lyophilized GNP-MAs or DBM-NP-MAs to create a thick paste. This mixture was then stirred vigorously with a spatula, centrifuged to form a gel, and then stored at 4 ⁰C. The nanoparticles within the inks were allowed to swell for 72 h with twice daily mixing and centrifugation to reduce aggregation.

*S1.2 3D printing of GNP-MA and DBM-NP-MA colloidal inks*

GNP-MA and DBM-NP-MA inks were extrusion 3DP on a BioAssemblyBot® 3D printer (Advanced Solutions Life Sciences). Both inks were printed using a 20 G needle and 0.8 mm/s print speed. GNP-MA inks were printed with a print pressure of 15-25 psi, while DBM-NP-MA inks required higher pressures of 25-35 psi. Single layer, 3.5 mm x 6 mm constructs were printed using three parallel fibers with a center-to-center fiber spacing of 2 mm and with no designed pores to minimize cell loss through macropores on cell seeding (**Figure S4**). After printing, GNP-MA and DBM-NP-MA constructs were photocrosslinked using a UV light flash box (Otoflash Post Curing Light Pulsing Unit, EnvisionTEC Inc., Gladbeck, Germany) which delivered ~60 mW/cm^2^ of 230-410 nm wavelength UV over 50 s using 500 flashes (3 J/cm^2^ UV).

*S1.3 Evaluation of 3DP GNP-MA and DBM-NP-MA construct* in vitro *osteogenic potential*

*S1.3.1 Construct cell seeding*

Human bone marrow-derived mesenchymal stem cells (hbMSCs) were purchased from RoosterBio (Lot #00257, sex: male, age: 23 years, CD34 and CD45 antigen: <10% positive, CD90 and CD166 antigen: >90% positive, Frederick, MD) and expanded in basal growth media [Advanced MEM (Invitrogen, Carlsbad, CA, USA), 10% fetal bovine serum, 1% GlutaMAX, and 1% anti-anti]. Prior to cell seeding, GNP-MA and DBM-NP-MA constructs were sterilized using ethylene oxide (12 h cycle; Anprolene AN74i; Anderson Sterilizers, Haw River, NC), allowed to vent for 48 h, and then swollen overnight in basal growth media. Using ultra-low attachment plates, constructs were seeded with 1.25 x 10^5^ hbMSCs. Constructs were then cultured in either basal growth media or osteogenic media (basal growth media supplemented with 50 mg/L ascorbic acid, 10^−8^ M dexamethasone, and 10 mM β‐glycerol 2‐phosphate). Media was exchanged after 24 h and then twice weekly over the course of 21 days. At days 3, 14, and 21 of culture, constructs in each group were collected and analyzed. Four constructs were used for each group (GNP-MA or DBM-NP-MA constructs in basal or osteogenic media) at each time point.

*S1.3.2 Biochemical analysis for osteogenesis*

At days 3, 14, and 21 of culture, corresponding constructs were washed with PBS for 15 min at 37 ⁰C and collected for analysis. Constructs were stored at -20 ⁰C until homogenization in 300 µL of sterile Milli-Q water with a Qiagen TissueLyser II (Hilden, Germany) at 30 s^-1^ for 5 min. 60 µL of each homogenized sample was combined 1:1 with a digestion buffer (2 mg/mL proteinase K, 20 μg/mL pepstatin A, and 370 μg/mL iodoacetamide in tris-EDTA solution (12.11 mg/mL tris(hydroxymethyl aminomethane), 0.744 mg/mL EDTA, pH 7.6) for degradation prior to analysis of DNA content. PicoGreen assay (Quant-iT™ 1X dsDNA Assay Kit, high sensitivity, Invitrogen, Carlsbad, CA) was performed according to manufacturer instructions to determine DNA content. Aliquots of 70 µL of homogenized samples were then combined 1:1 with 1 M acetic acid and incubated overnight at room temperature with agitation to extract Ca^2+^ ions for quantification. Calcium content was then assessed using a colorimetric Arsenazo III kit (Pointe Scientific, Canton, MI) according to previously published methods.15 Briefly, 30 µL of each sample was incubated with 75 µL of Arsenazo III reagent for 10 min prior to reading absorbance values at 650 nm using a plate reader (Powerwave x340 Microplate Reader, BioTek Instruments, VT).

**Supplementary Figures**


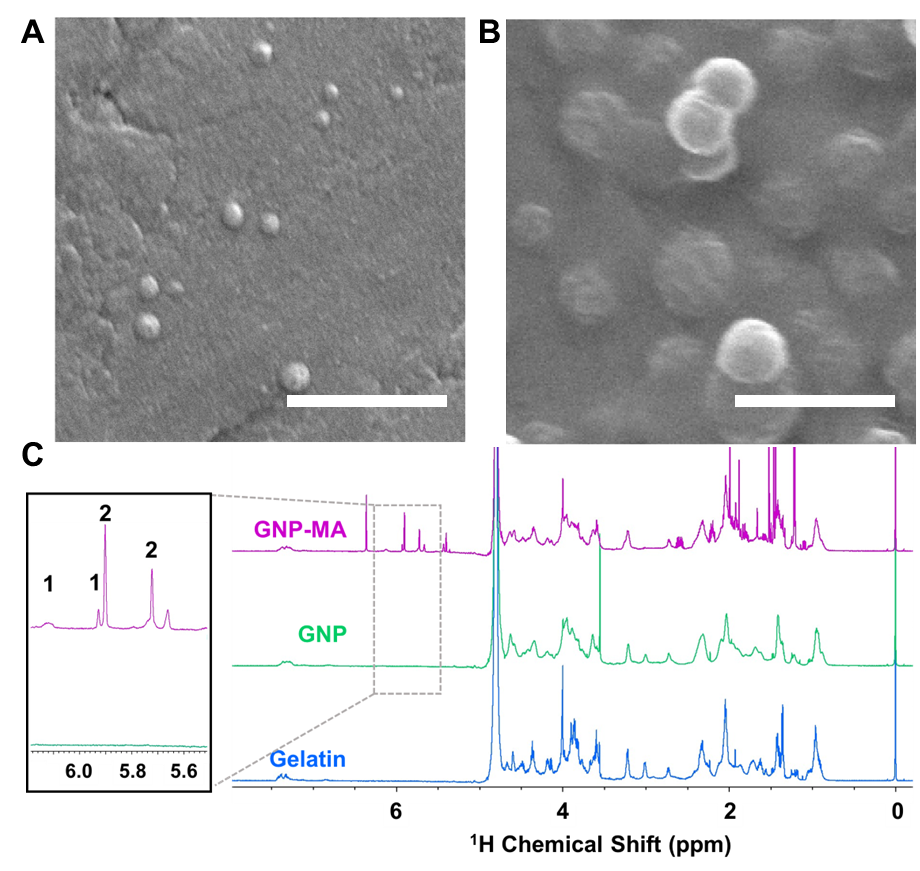


**Figure S1**. Characterization of GNPs and GNP-MAs. Representative SEM images of freeze-dried (A) GNPs and (B) GNP-MAs. (C) ^1^H NMR spectra of gelatin, GNPs, and GNP-MAs for confirmation of methacryloylation: methacrylate (CH_2_=C(CH_3_)COO-) peaks labeled 1 (5.9-6.2 ppm) and methacrylamide (CH_2_=C(CH_3_)CONH-) peaks labeled 2 (5.6-5.9 ppm). Scale bar = 500 nm. GNPs, gelatin nanoparticles; GNP-MAs, methacryloylated gelatin nanoparticles.


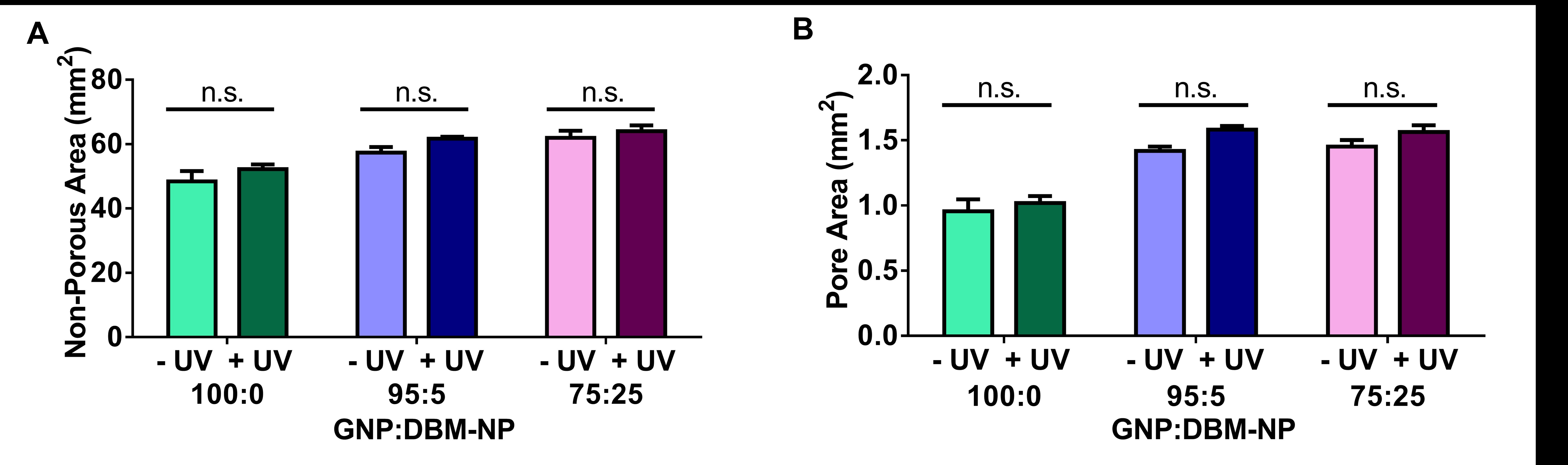


**Figure S2**. Composite GNP:DBM-NP construct morphological changes with UV exposure. Construct (E) non-porous area and (F) pore area before and after UV crosslinking with 3 J/cm^2^. Error bars represent standard deviation. n.s. indicates non-significance (n = 4, p < 0.05).


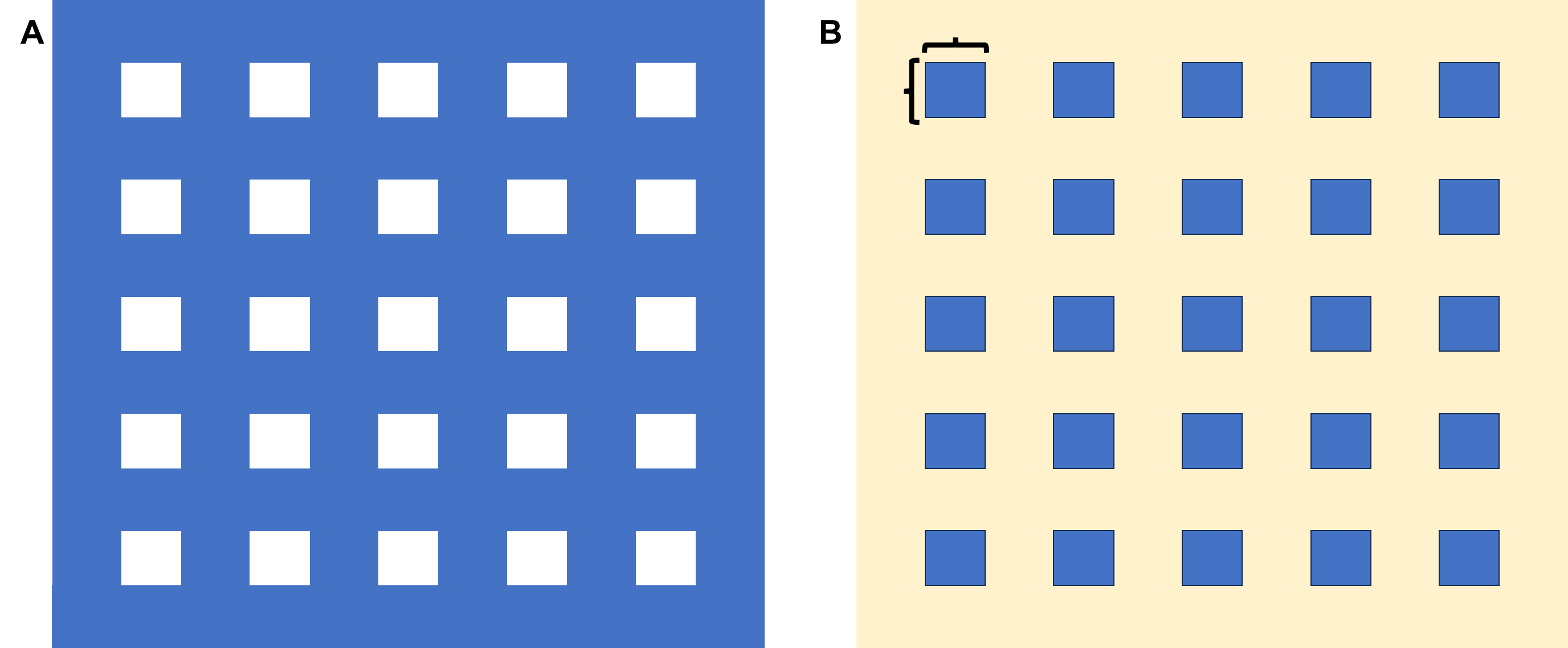


**Figure S3.** Schematic of construct (A) non-porous area and (B) pore area quantified in swelling data (shown in blue). All values are normalized to initial post-printing values. Pore area refers to the average of individual pore area for all pores maintained within the construct.


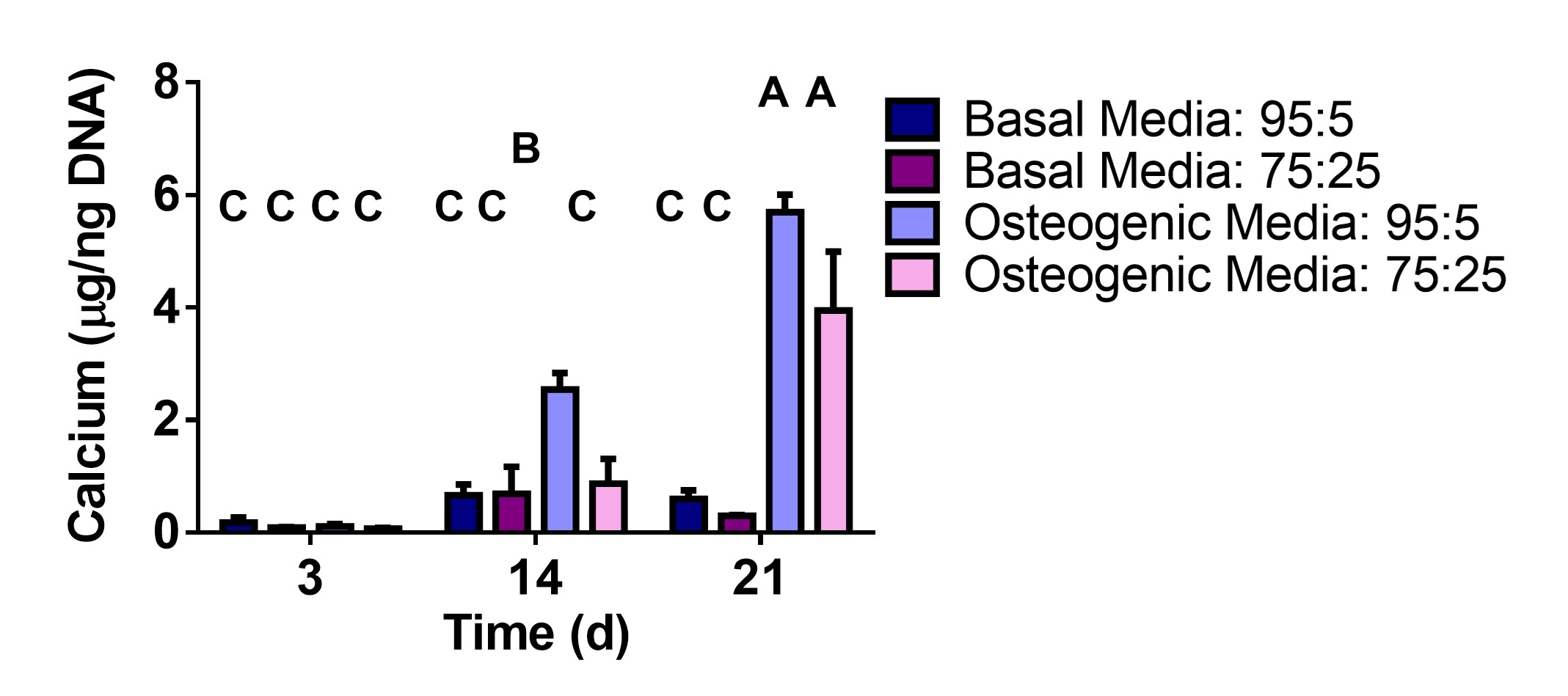


**Figure S4.** Calcium content normalized by DNA content quantified via Arsenazo III assay for 95:5 and 75:25 GNP:DBM-NP constructs seeded with human bone marrow-derived mesenchymal stem cells relative to DBM-NP-MA content and culture media type. Shared letters indicate no significant difference between groups. Error bars represent standard deviation (n = 3-4, p < 0.05).


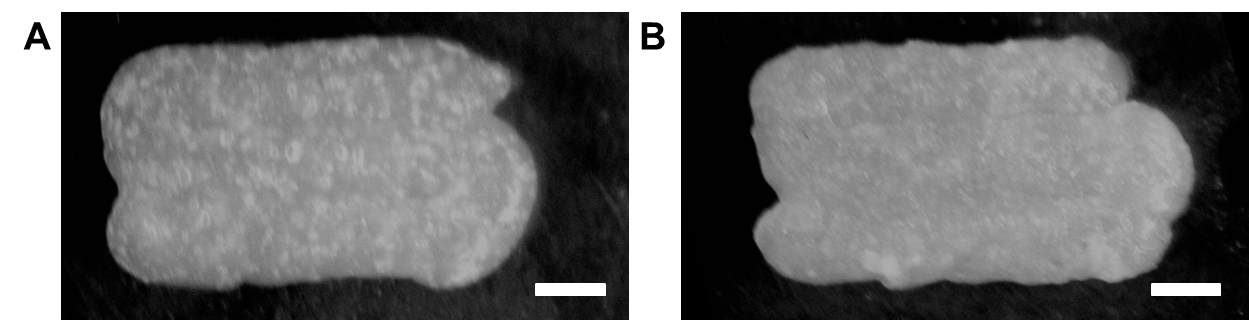


**Figure S5.** Representative light microscopy images of 3DP constructs composed of A) GNP-MAs and B) DBM-NP-MAs. Scale bar = 1 mm.


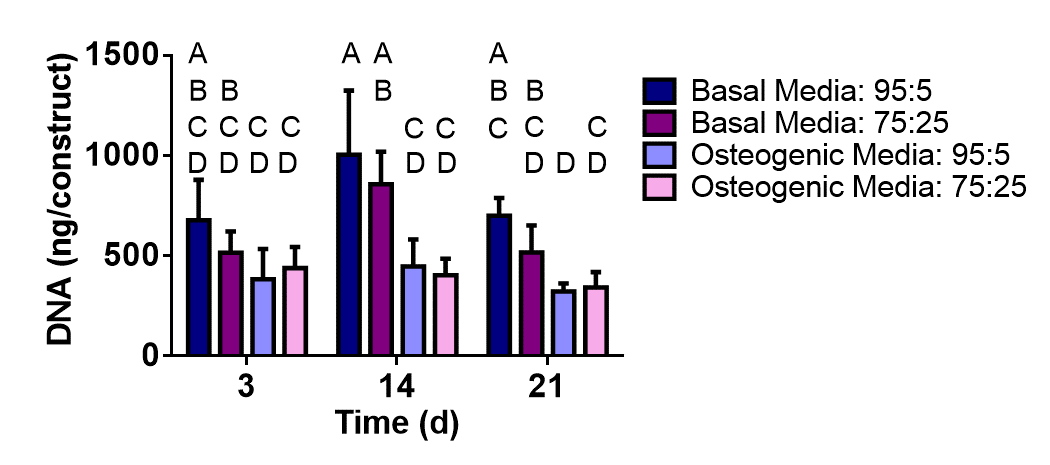


**Figure S6.** DNA content per construct quantified via PicoGreen assay for DBM-NP-MA-only and GNP-MA-only constructs seeded with human bone marrow-derived mesenchymal stem cells relative to culture media type. Shared letters indicate no significant difference between groups. Error bars represent standard deviation (n = 4, p < 0.05).


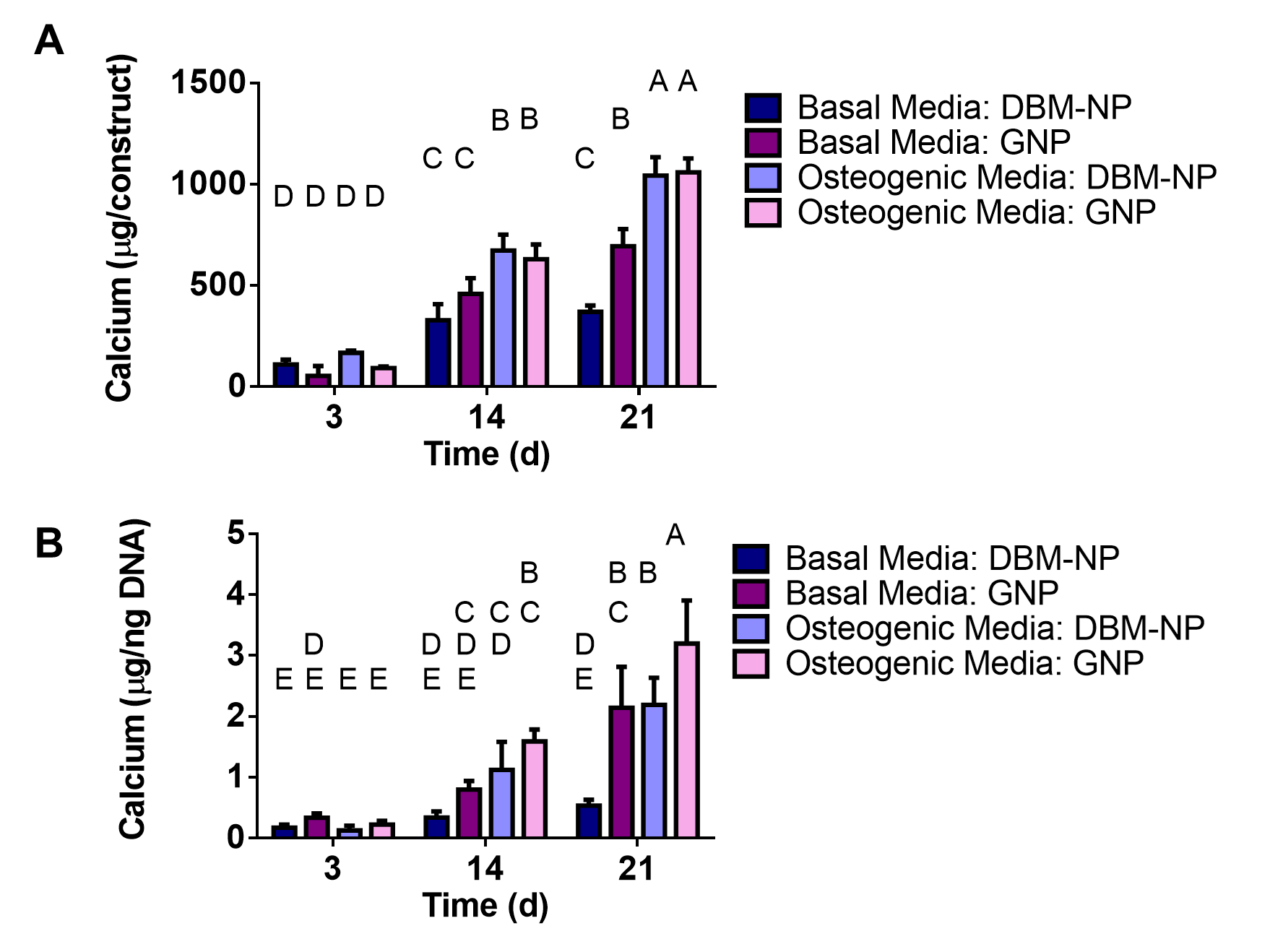


**Figure S7.** Calcium content (A) per construct and (B) normalized by DNA content quantified via Arsenazo III assay for DBM-NP-MA-only and GNP-MA-only constructs seeded with human bone marrow-derived mesenchymal stem cells relative to culture media type. Shared letters indicate no significant difference between groups. Error bars represent standard deviation (n = 4, p < 0.05).
